# Gene pyramiding for boosted plant growth and broad abiotic stress tolerance

**DOI:** 10.1111/pbi.14216

**Published:** 2023-10-30

**Authors:** Guiqin Zhao, Yu Liu, Lei Li, Rui Che, Megan Douglass, Katherine Benza, Mitchell Angove, Kristopher Luo, Qian Hu, Xiaotong Chen, Charles Henry, Zhigang Li, Guogui Ning, Hong Luo

**Affiliations:** ^1^ Department of Genetics and Biochemistry Clemson University Clemson SC USA; ^2^ College of Grassland Science Gansu Agricultural University Lanzhou Gansu China; ^3^ College of Landscape Architecture Northeast Forestry University Harbin Heilongjiang China; ^4^ College of Agronomy Henan Agricultural University Zhengzhou Henan China; ^5^ Key laboratory of Horticultural Plant Biology, Ministry of Education Huazhong Agricultural University Wuhan China

**Keywords:** gene pyramiding, synergistic effect, abiotic stress, drought tolerance, salt tolerance, perennial grasses

## Abstract

Abiotic stresses such as salinity, heat and drought seriously impair plant growth and development, causing a significant loss in crop yield and ornamental value. Biotechnology approaches manipulating specific genes prove to be effective strategies in crop trait modification. The *Arabidopsis* vacuolar pyrophosphatase gene *AVP1*, the rice SUMO E3 ligase gene *OsSIZ1* and the cyanobacterium flavodoxin gene *Fld* have previously been implicated in regulating plant stress responses and conferring enhanced tolerance to different abiotic stresses when individually overexpressed in various plant species. We have explored the feasibility of combining multiple favourable traits brought by individual genes to acquire superior plant performance. To this end, we have simultaneously introduced *AVP1*, *OsSIZ1* and *Fld* in creeping bentgrass. Transgenic (TG) plants overexpressing these three genes performed significantly better than wild type controls and the TGs expressing individual genes under both normal and various abiotic stress conditions, exhibited significantly enhanced plant growth and tolerance to drought, salinity and heat stresses as well as nitrogen and phosphate starvation, which were associated with altered physiological and biochemical characteristics and delicately fine‐tuned expression of genes involved in plant stress responses. Our results suggest that AVP1, OsSIZ1 and Fld function synergistically to regulate plant development and plant stress response, leading to superior overall performance under both normal and adverse environments. The information obtained provides new insights into gene stacking as an effective approach for plant genetic engineering. A similar strategy can be extended for the use of other beneficial genes in various crop species for trait modifications, enhancing agricultural production.

## Introduction

Dramatic climate changes in recent decades have altered global environments, creating an increasing challenge for agricultural practice and production (Alkorta *et al*., 2017; Cohen *et al*., 2021; Hamann *et al*., 2021; Lobell and Gourdji, 2012; Ray *et al*., 2019; Rivero *et al*., 2022; Salih *et al*., 2020; Zandalinas *et al*., 2021a,b). Drought, salinity, heat and nutrient scarcity are among the main stresses significantly affecting plant growth and crop productivity. The rapid development of resilient and tailored crop varieties is critical in maintaining food security under a rapidly changing climate. With the development of recombinant DNA and gene transformation technologies, plant genetic improvement using effective biotechnology approaches to manipulate target genes and biological pathways in TGs for enhanced performance and production under various environmental adversities has long been successfully demonstrated in model species and many important crops (Mittler and Blumwald, 2010; Varshney *et al*., 2011), and is becoming an important and integrated part of the modern agriculture practice.

*Arabidopsis thaliana* vacuolar H^+^‐pyrophosphatase gene, *AVP1* and its homologues from different sources are among the widely used candidates for manipulation in genetic engineering of salinity and drought tolerance in plants such as *Arabidopsis* (Gaxiola *et al*., 2001), tobacco (Duan *et al*., 2007), tomato (Park *et al*., 2005), cotton (Asad *et al*., 2008; Lv *et al*., 2008, 2009), corn (Li *et al*., 2008) and creeping bentgrass (Li *et al*., 2010). The AVP1 TG *Arabidopsis*, rice and tomato also exhibited improved phosphorus uptake (Yang *et al*., 2007). To improve plant tolerance to drought, many other genes have been demonstrated to play essential roles, including those for a rice SUMO E3 ligase OsSIZ1 (Li *et al*., 2013; Mishra *et al*., 2017), a cyanobacterial flavodoxin Fld (Li *et al*., 2017), sodium/hydrogen antiporters (Huang *et al*., 2018b; Long *et al*., 2020), protein kinases (Masle *et al*., 2005; Saijo *et al*., 2000), various transcription factors (Agarwal *et al*., 2017; Chen *et al*., 2007; Fernando *et al*., 2021; Hu *et al*., 2006; Huang *et al*., 2018a; Peng *et al*., 2011; Sarkar *et al*., 2014; Selvaraj *et al*., 2020; Yang *et al*., 2020; Zhou *et al*., 2016), LEA and 14‐3‐3 proteins (Bahieldin *et al*., 2005; Fu *et al*., 2007; Lal *et al*., 2008; Maqbool *et al*., 2002; Rohila *et al*., 2002; Xiao *et al*., 2007; Xu *et al*., 1996; Yan *et al*., 2004), biosynthesis of oligosaccharides and proline (Abebe *et al*., 2003; Fernando *et al*., 2021; Garg *et al*., 2002; Kishor *et al*., 1995; Selvaraj *et al*., 2017; Zhu *et al*., 1998) and ABA signalling (Ito *et al*., 2006; Oh *et al*., 2005; Vishwakarma *et al*., 2017; Wang *et al*., 2003). Similar strategies have also been used to improve plant performance under heat, which is another abiotic stress of increasing importance due to global warming affecting plant growth and development. So far, TG plants with modified expression of specific target genes that exhibit enhanced heat tolerance have been reported in various species including *Arabidopsis* (Chauhan *et al*., 2013; Guo *et al*., 2015; He *et al*., 2016; Queitsch *et al*., 2000; Singh and Khurana, 2016; Zhang *et al*., 2013, 2017), tobacco (Murakami *et al*., 2000; Ono *et al*., 2001; Sanmiya *et al*., 2004; Yang *et al*., 2005), rice (Qin *et al*., 2015; Wang *et al*., 2012), tomato (Cheng *et al*., 2009; Li *et al*., 2003a; Wang *et al*., 2006), wheat (Hu *et al*., 2018; Xue *et al*., 2015; Zhang *et al*., 2017) and creeping bentgrass (Li *et al*., 2013, 2017; Zhao *et al*., 2018).

However, plants growing in harsh environments often simultaneously face multiple stresses such as salinity, drought, heat and nutrient starvation. Improving one trait or feature through genetic modification of a single candidate gene is insufficient in increasing overall plant performance and productivity (Rivero *et al*., 2022). To this end, more genes or pathways usually need to be manipulated simultaneously in TG plants to alter multiple traits, thus producing significantly improved new cultivars able to cope with complex environmental adversities. Similarly, metabolic engineering also often requires the manipulation of various genes implicated in specific biological pathways. Multigene manipulation for trait modifications has previously been reported in both model and crop species, including *Arabidopsis* (Esmaeili *et al*., 2019; Slater *et al*., 1999), tobacco (Duan *et al*., 2009; Singla‐Pareek *et al*., 2003), corn (Bohorova *et al*., 1999), rice (Chen *et al*., 1998; Hur *et al*., 2016; Ye *et al*., 2000), cotton (Esmaeili *et al*., 2021; Shen *et al*., 2014), pepper (Zhu *et al*., 2015), aspen (Li *et al*., 2003b) and banana (Ghislain *et al*., 2019). However, due to the potentially differential interactions or crosstalk between genes functioning in the same or different biological or regulatory pathways in plants of diverse genetic background, there might be gene silence, multigene antagonism, positive or negative epistasis taking place when implementing multigene manipulation strategies for trait modifications to achieve plant resistance to a plethora of complex environmental stresses (Anderson *et al*., 2004; Boer *et al*., 2014; González‐Reig *et al*., 2012; Hepworth *et al*., 2002; Miedaner *et al*., 2006; Takahashi *et al*., 2004; Yamamichi *et al*., 2019; Zhuang *et al*., 2002).

Creeping bentgrass (*Agrostis stolonifera*) is a perennial cool season turfgrass that provides numerous environmental, societal and economic benefits (Haydu *et al*., 2006). Its improved performance under diverse environmental adversities would reduce establishment and maintenance costs, expand potential growth areas and benefit the environment. To this end, we have adopted biotechnology approaches to manipulate the expression of individual structural and regulatory protein genes, as well as non‐coding small RNA genes in TGs to produce new creeping bentgrass materials with altered plant development and enhanced plant tolerance to various abiotic stresses (Li *et al*., 2010, 2013, 2017; Sun *et al*., 2021; Yuan *et al*., 2015, 2019, 2020; Zhao *et al*., 2018; Zhou *et al*., 2013). Particularly, we showed that TG creeping bentgrass overexpressing an *Arabidopsis* H^+^‐pyrophosphatase gene, *AVP1* significantly enhanced plant salt tolerance compared to wild type (WT) controls (Li *et al*., 2010). Constitutive expression of the H^+^‐pyrophosphatase gene incurred not only an altered sodium sequestration but also a modified auxin transport and accumulation regulating organogenesis. As a result, overexpression of the *AVP1* gene enhanced root and shoot growth through facilitated auxin flux (Li *et al*., 2005, 2010). We also displayed that introducing a rice SUMO E3 ligase gene, *OsSIZ1* into creeping bentgrass led to enhanced plant drought tolerance (Li *et al*., 2013). Sumoylation is a posttranslational regulatory process that modifies substrate proteins through the conjugation of small ubiquitin‐related modifiers (SUMOs). SUMO E3 ligase, a critical component in the sumoylation pathway, regulates diverse plant processes in development and stress responses (Elrouby and Coupland, 2010; Miller *et al*., 2010; Miller and Vierstra, 2011; Park *et al*., 2011a,b; Reed *et al*., 2010). The increased drought tolerance in the *OsSIZ1*‐overexpressing creeping bentgrass was associated with more robust root growth, higher water retention and a better cell membrane integrity than WT controls. The TG plants also exhibited better plant growth under phosphate (Pi) starvation conditions than WT controls (Li *et al*., 2013). Flavodoxin (Fld) plays an essential role in photosynthetic microorganisms as an alternative electron carrier flavoprotein under adverse environmental conditions (Singh *et al*., 2004; Yousef *et al*., 2003; Zheng *et al*., 1999), and cyanobacterial Fld was able to substitute ferredoxin of higher plants in most electron transfer processes under abiotic stress (Blanco *et al*., 2011; Ceccoli *et al*., 2011, 2012; Tognetti *et al*., 2006, 2007a,b). We generated TG creeping bentgrass constitutively expressing a cyanobacterial *Fld* gene. The *Fld* TG plants exhibited a significantly improved performance under heat stress and nitrogen starvation conditions, which was associated with changes in water retention, cell membrane integrity, expression of heat‐shock protein genes and increased accumulations of more reduced thioredoxin, nitrogen (N) and total chlorophyll when compared with WT controls (Li *et al*., 2017). However, overexpression of *Fld* negatively impacted plant development, causing delayed plant growth and significantly reduced biomass production in *Fld* TGs (Li *et al*., 2017).

These three genes, *AVP1*, *OsSIZ1* and *Fld* encode proteins representing different mechanisms of stress response regulation in plants. When individually manipulated in TG plants, each one contributes to certain aspects of plant stress tolerance. Two of them, *OsSIZ1* and *AVP1*, when co‐overexpressed in *Arabidopsis*, rendered enhanced plant tolerance to drought, salinity and heat stress (Esmaeili *et al*., 2019). Their co‐overexpression in cotton led to a significantly improved plant performance under single and multiple stress conditions in laboratory and field conditions compared to *AVP1*‐overexpressing, *OsSIZ1*‐overexpressing and WT control plants (Esmaeili *et al*., 2021). Two field studies showed that OsSIZ1/AVP1 co‐overexpressing plants produced 133% and 81% more fibre as compared to WT cotton in the dryland conditions of West Texas (Esmaeili *et al*., 2021). These results suggest the feasibility of simultaneously manipulating two genes with completely different functioning mechanisms to combine their benefits to produce TG plants with desired multiple traits rendered by both genes. However, whether a similar strategy could be applied to important monocot crops, especially perennial grass species remain unclear. Moreover, although many genes, such as *AVP1, OsSIZ1* and *Fld*, function to positively regulate various biological pathways beneficial to plant growth and/or stress responses, they may also act to adversely impact plants leading to agronomically undesirable traits as observed in *Fld* TGs described above (Li *et al*., 2017). This raises the question of whether gene pyramiding could provide a remedy to this dilemma, specifically, could the negative effects by each one of the stacked genes be compensated by features from the others? The current study sought to address these questions by co‐expressing three different stress‐related genes encoding an *Arabidopsis* vacuolar H^+^‐pyrophosphatase AVP1, a rice SUMO E3 ligase OsSIZ, and a cyanobacterial flavodoxin Fld in TG creeping bentgrass. Our results suggest that AVP1, OsSIZ1 and Fld synergistically regulate plant development and stress response, leading to superior overall performance under both normal and adverse environments. The data obtained also indicate that undesirable adverse effects by individual genes can be effectively compensated through gene pyramiding to maximize beneficial characteristics leading to significantly improved overall plant performance in development and stress responses. Similar approaches may be used to manipulate other candidate genes to control any traits of interest in plants. The data obtained provide new insights into gene stacking as an effective approach for plant genetic engineering. This strategy could be adapted and extended to use other beneficial genes in various crops species for trait modifications, enhancing agricultural production.

## Results

### Generation and molecular characterization of TG creeping bentgrass plants expressing *AVP1*, *OsSIZ1* and *Fld*

**Figure 1:**
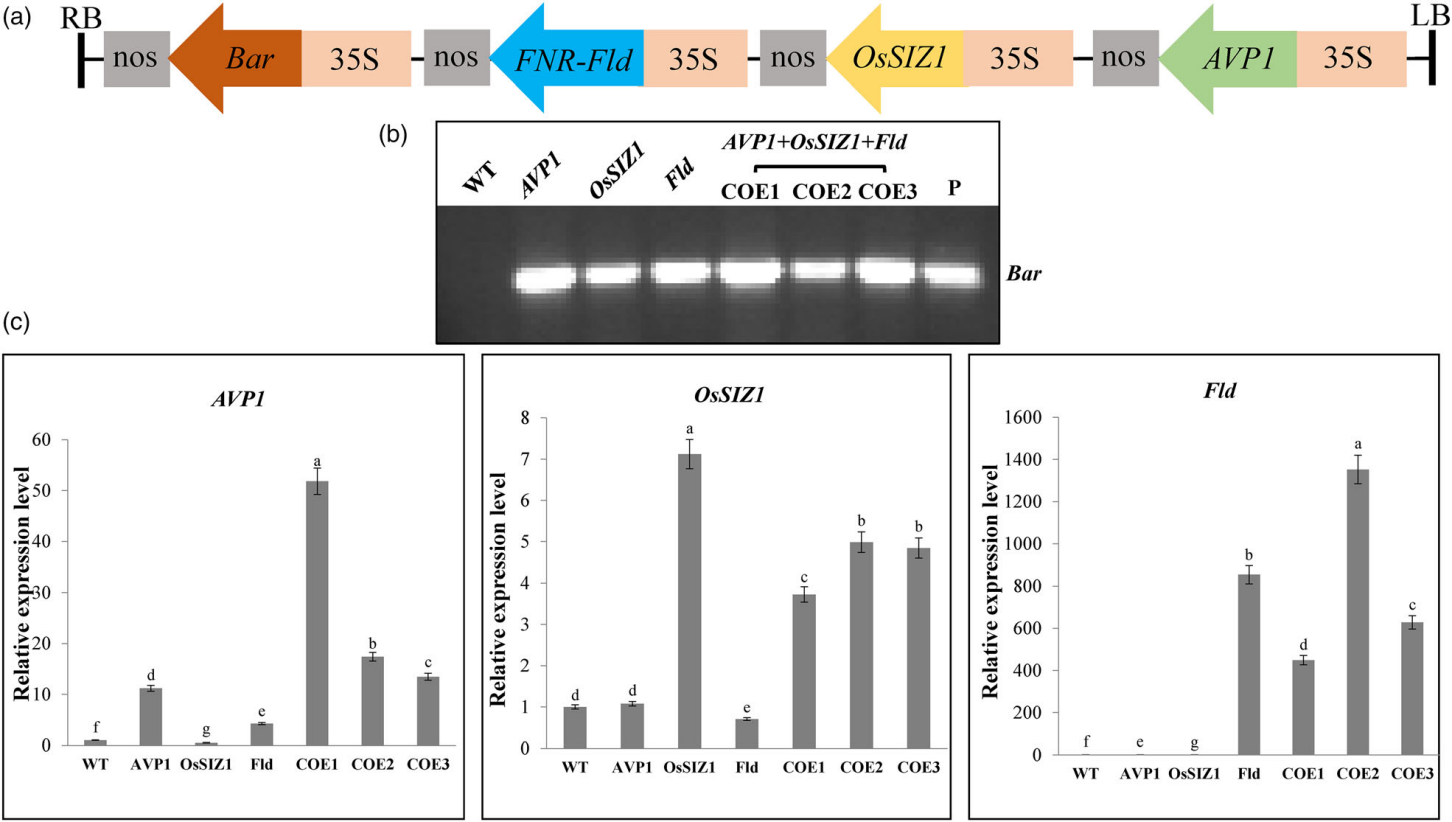
Overexpression of *AVP1*, *OsSIZ1* and *Fld* in transgenic (TG) creeping bentgrass plants. (a) Schematic diagram of the *AVP1*, *OsSIZ1* and *Fld* chimeric gene expression construct, p35S‐AVP1/35S‐OsSIZ1/35S‐Fld/35S‐bar. The three genes, *AVP1*, *OsSIZ1* and *Fld* are all under the control of the cauliflower mosaic virus 35S gene promoter and linked to the herbicide resistance gene, *bar*, also driven by the CaMV 35S promoter. (b) Detection of *AVP1*, *OsSIZ1* and *Fld* in TG creeping bentgrass plants. Genomic DNA was extracted from 1 g of young leaves of three representative TG lines (COE1‐3) harbouring p35S‐AVP1/35S‐OsSIZ1/35S‐Fld/35S‐bar, non‐TG wild type (WT) plants and additional three previously generated control TG lines containing *AVP1* (Li *et al*., 2010), *OsSIZ1* (Li *et al*., 2013) and *Fld* (Li *et al*., 2017), respectively. Transgene presence was determined by PCR on genomic DNA to amplify the *bar* gene. Plasmid DNA (P) of the construct, p35S‐AVP1/35S‐OsSIZ1/35S‐Fld/35S‐bar was used as a positive control. PCR products were run on a 1.5% (w/v) agarose gel and stained with ethidium bromide. (c) Expression of *AVP1*, *OsSIZ1* and *Fld* in TG creeping bentgrass plants. Total RNA was extracted from 1 g of young leaves of TG lines expressing all three genes, *AVP1, OsSIZ1* and *Fld* (COE1‐3) or only one of them, *AVP1* (Li *et al*., 2010), *OsSIZ1* (Li *et al*., 2013) or *Fld* (Li *et al*., 2017) as well as the non‐TG WT controls. Transgene expression was determined by RT–PCR on cDNA to amplify *AVP1, OsSIZ1* and *Fld*. The ΔΔCt method was used to analyse the relative gene expression levels. *Actin* was used as an endogenous control. Data are presented as means of three biological replicates × three technical replicates; error bars represent standard deviation. Statistically significant differences between various plant lines were determined by one‐way ANOVA. Posthoc comparisons using Tukey's honestly significant difference (HSD) test were conducted to determine the overall difference between groups. Means not sharing the same letter are statistically significantly different (*P* < 0.05).

To explore the potentially additive effect of multiple stacked genes involved in regulating plant development and stress responses, we transformed creeping bentgrass plants with a chimeric gene construct containing *AVP1*, *OsSIZ1* and *Fld* (Figure 1a) to produce a total of 17 independent TG lines (see examples in Figure 1b). RT–PCR analysis revealed the high‐level expression of *AVP1*, *OsSIZ1* and *Fld* in all TG lines (see examples in Figure 1c). All TG lines grown and assessed in the greenhouse were phenotypically indistinguishable and exhibited no difference in response to various cultivation conditions. Three representative lines, COE1, COE2 and COE3, containing a single copy of transgene expression cassette, *AVP1/OsSIZ1/Fld*, were chosen for further analysis (Table S1). Non‐TG creeping bentgrass plants and three representative TG lines each harbouring and expressing a single transgene of either *AVP1*, *OsSIZ1* or *Fld* that had previously been characterized (Li *et al*., 2010, 2013, 2017) were also included in this study as controls (Figure 1b,c). The WT and selected TG lines were all clonally multiplied by vegetative propagation and maintained in greenhouse for analysis.

### Co‐overexpression of *AVP1*, *OsSIZ1* and *Fld* significantly improves plant growth and development

**Figure 2:**
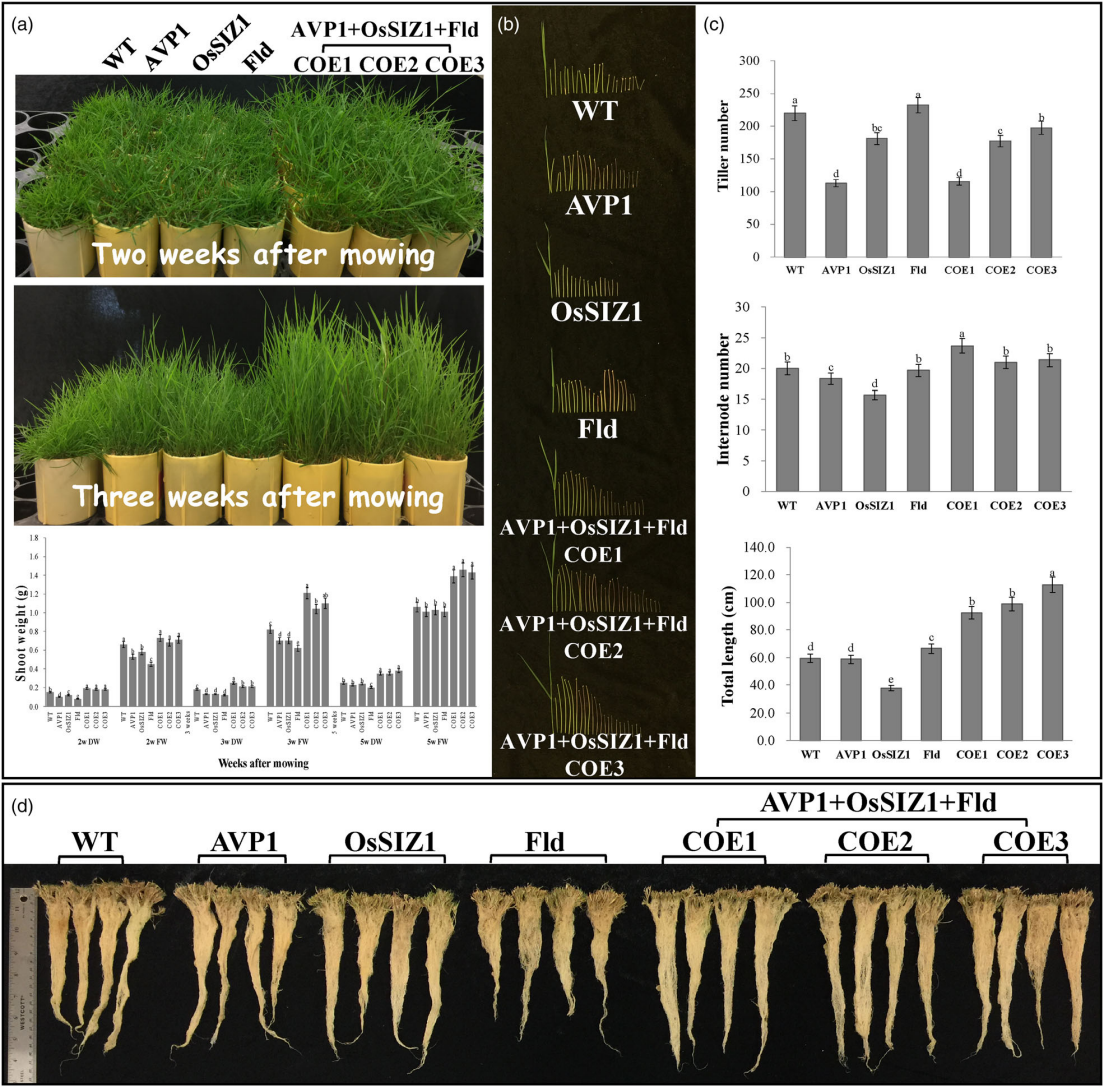
Plant growth and development in TG and WT controls. (a) TG lines expressing all three transgenes, *AVP1, OsSIZ1* and *Fld* (TG1‐3) or only one of them, *AVP1* (Li *et al*., 2010), *OsSIZ1* (Li *et al*., 2013) or *Fld* (Li *et al*., 2017) as well as the non‐TG WT controls initiated from the same number of tillers were fully developed in cone‐taines for 10 weeks under normal conditions in growth room. The 10 weeks old plants were then uniformly trimmed and grown for additional 2 (top panel) or 3 weeks (middle panel) before measuring their shoot fresh weight (FW) and dry weight (DW) (bottom panel). (b) All internodes from the representative longest tiller of each plant line tested were sliced from top to bottom and displayed in order from left to right. (c) The total tillers number (top), the total internodes number (middle) of the longest tiller and the total length of the longest tiller (bottom) from the representatives of WT control plants and various TG lines tested. Data are shown as means (*n* = 3); error bars represent standard errors. Statistically significant differences between various plant lines were determined by one‐way ANOVA. Posthoc comparisons using Tukey's HSD test were conducted to determine the overall difference between groups. Means not sharing the same letter are statistically significantly different (*P* < 0.05). (d) Root development in 13‐week‐old plants of the various TG lines and WT controls.

**Figure 3:**
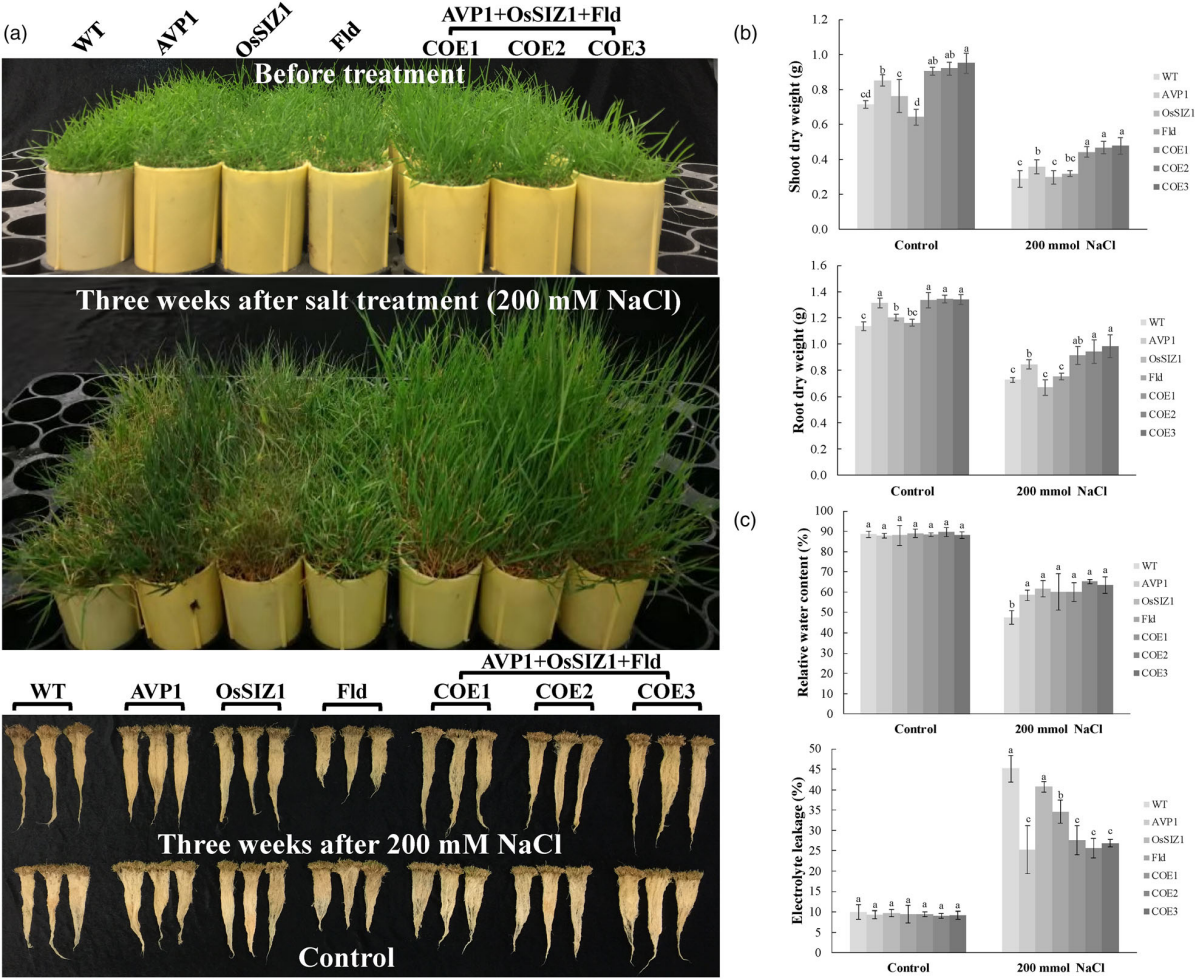
Plant salt stress responses in various TG lines and WT control plants. (a) The TG lines expressing all three transgenes, *AVP1, OsSIZ1* and *Fld* (TG1‐3) or only one of them, *AVP1* (Li *et al*., 2010), *OsSIZ1* (Li *et al*., 2013) or *Fld* (Li *et al*., 2017) as well as the non‐TG WT controls initiated from the same number of tillers were fully developed in cone‐tainers for 10 weeks under normal conditions in growth room. The 10 weeks old plants were then uniformly trimmed and grown for one more week (top panel) before being subjected to 3 weeks of 200 mM NaCl treatment (middle panel). The bottom panel shows plant root development under salt stress and normal conditions without treatment. (b) Shoot and root DWs of the WT controls and various TG lines 3 weeks after 0 and 200 mM NaCl treatment. (c) Leaf relative water content (RWC) and electrolyte leakage (EL) of the WT controls and various TG lines were measured before and 3 weeks after 200 mM NaCl treatment. Data are shown as means (*n* = 3); error bars represent standard deviation. Statistically significant differences between various plant lines were determined by one‐way ANOVA. Posthoc comparisons using Tukey's HSD test were conducted to determine the overall difference between groups. Means not sharing the same letter are statistically significantly different (*P* < 0.05).

To examine how gene pyramiding would impact overall plant growth and development, we compared TG creeping bentgrass plants expressing all three transgenes, *AVP1*, *OsSIZ1* and *Fld* to those expressing either *AVP1*, *OsSIZ1* or *Fld* and the WT controls. Figure 2a shows examples of the various test lines grown for 2–5 weeks from the uniformly trimmed 8‐week‐old plants. The TG plants with all three transgenes exhibited a more vigorous shoot growth with a significantly higher biomass production than the WT controls and the TG plants expressing only one of the three transgenes. The average dry weight (DW) of the TG plants expressing all three transgenes was 45.16% higher than that of WT, and 58.59%, 52.54% and 80.00% higher than that of *AVP1*, *OsSIZ1* and *Fld* TG plants, respectively (Figure 2a). The enhanced shoot growth in the TG plants expressing all three transgenes did not seem to be associated with tiller or internode number but rather with extended internode length (Figure 2b,c). Additionally, the TG plants expressing all three transgenes also exhibited a more robust root system than both the WT controls and the *AVP1*, *OsSIZ1* and *Fld* TG lines (Figures 2d, 3a,b and 7b). It should be noted that although the TG lines expressing *AVP1* or *OsSIZ1* exhibited significantly higher shoot biomass production compared to WT controls under stressful conditions (Figures 3, 5, 6 and 7), they did not show significant differences under normal conditions (Figure 2) as previously observed (Li *et al*., 2010, 2013), most likely due to the difference in plant culture conditions. The TG plants grown in potting mixture soil (Fafard 3‐B Mix, Fafard Inc., Anderson, SC) in this study may not be able to rapidly attain their maximal growth potential compared to those grown in sand with supplied nutrients as reported previously (Li *et al*., 2010, 2013).

### Co‐overexpression of *AVP1*, *OsSIZ1* and *Fld* improves plant tolerance to salinity stress

To investigate how the various TG lines perform under salinity stress, we applied nutrient solution supplemented with 200 mM NaCl to well‐maintained WT and all TG lines daily for 3 weeks, and then compared plant responses. We observed that while nearly all TG lines exhibited a more vigorous shoot and root growth with a significantly higher biomass production than the WT control plants under normal growth conditions (Figure 3a,b) as already demonstrated above (Figure 2a–c), both WT and TG lines displayed salt‐elicited symptoms upon salinity stress, but with different levels of severity (Figure 3a). The TG plants expressing all three transgenes, *AVP1*, *OsSIZ1* and *Fld* exhibited the least damage (leaf chlorosis and retarded plant growth), followed by the TG plants expressing *AVP1* and those expressing *Fld*. In contrast, WT and TG plants expressing *OsSIZ1* displayed the most severe damage (Figure 3a,b). The shoot biomass of the TG plants expressing all three transgenes, *AVP1*, *OsSIZ1* and *Fld* remained the highest, followed by the TG plants expressing *AVP1* and those expressing *Fld*, whereas WT and TG plants expressing *OsSIZ1* had the lowest shoot biomass (Figure 3a,b). A similar phenomenon was also observed in roots. Despite the reduced root growth resulting in DW loss in all plants tested under salt stress, root biomass of the TG plants expressing all three transgenes, *AVP1*, *OsSIZ1* and *Fld* remained the highest, followed by the TG plants expressing *AVP1*, those expressing *Fld*, WT and TG plants expressing *OsSIZ1* (Figure 3a,b). Accordingly, the salt‐inflicted water loss and the cell membrane damage measured as electrolyte leakage (EL) in all TG lines were significantly less severe than in the WT controls (Figure 3c), suggesting an enhanced capacity in water retention and cell membrane integrity maintenance in the TG plants compared with the WT controls, most notably in those expressing all three transgenes, *AVP1*, *OsSIZ1* and *Fld*, and those expressing only *AVP1* or *Fld*.

### TGs co‐expressing *AVP1*, *OsSIZ1* and *Fld* accumulate less Na^+^ in shoot, but more K^+^ in roots than controls and exhibit altered Ca^++^ uptake under salinity conditions

**Figure 4:**
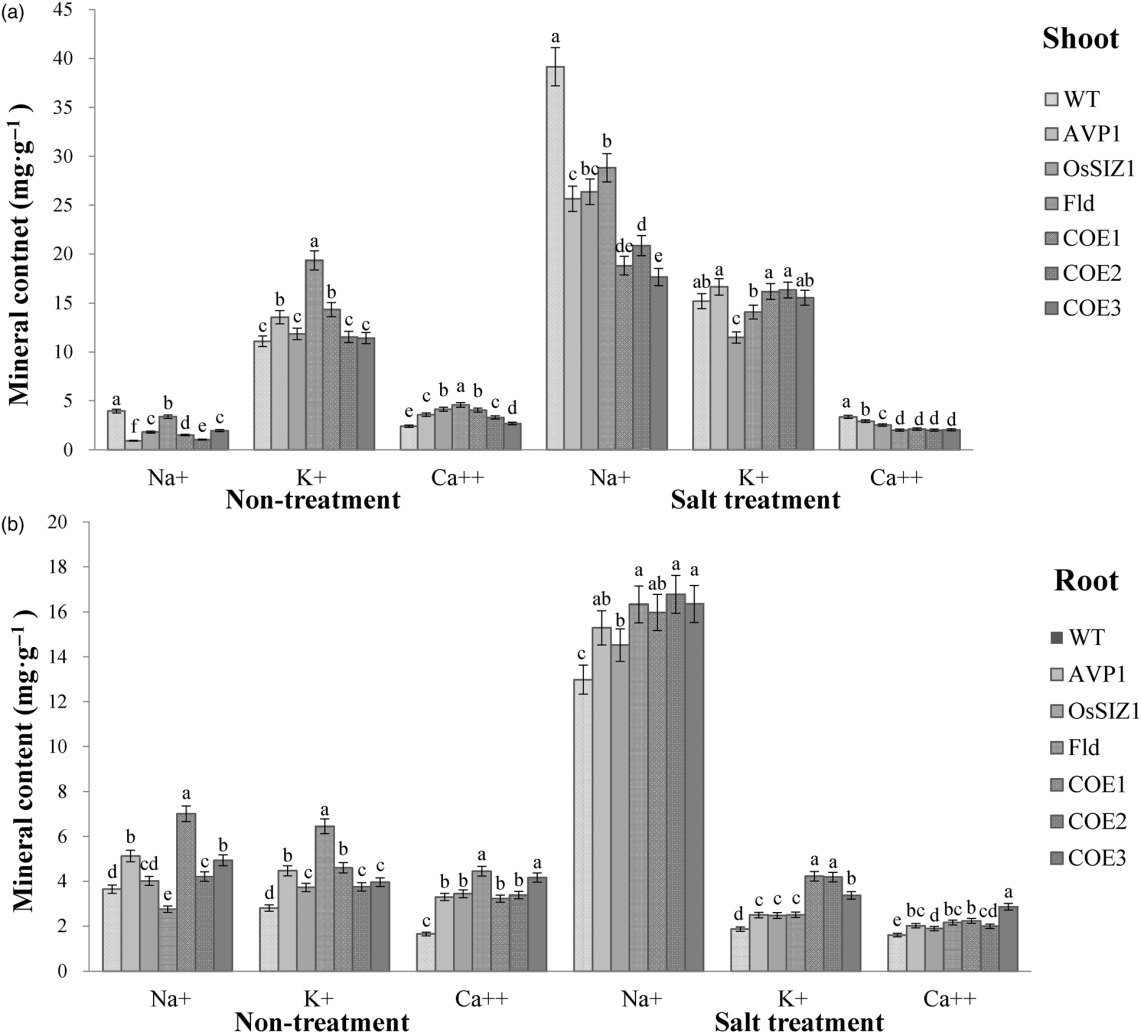
Shoot and root sodium (Na^+^), potassium (K^+^) and calcium (Ca^++^) contents in WT and TG lines under normal and salt stress conditions. Data are shown as means (*n* = 3); error bars represent standard deviation. Statistically significant differences between various plant lines were determined by one‐way ANOVA. Posthoc comparisons using Tukey's HSD test were conducted to determine the overall difference between groups. Means not sharing the same letter are statistically significantly different (*P* < 0.05).

To elucidate mechanisms underlying enhanced salt tolerance in the TG plants expressing all three transgenes, *AVP1*, *OsSIZ1* and *Fld*, and those expressing only *AVP1*, *OsSIZ1* or *Fld*, we measured sodium (Na^+^), potassium (K^+^) and calcium (Ca^++^) contents in all plant lines under normal and salt‐stressed conditions. As shown in Figure 4a, despite a significantly increased sodium uptake in all plant lines tested under salt treatment, every TG line had a significantly lower shoot sodium accumulation than the WT controls under both normal and salinity stress conditions. When subjected to salt stress, the TG plants expressing all three transgenes, *AVP1*, *OsSIZ1* and *Fld* had the lowest shoot sodium accumulation, followed by the TG plants expressing only *AVP1*, *OsSIZ1* or *Fld*. WT plants accumulated significantly more sodium in the shoots compared to TG lines (Figure 4a). In roots, nearly all TG plants, except the *OsSIZ1* and *Fld* TG lines grown under normal conditions, exhibited significantly higher sodium accumulations than WT controls under both normal and salt stress conditions (Figure 4a).

K^+^ is an essential mineral that functions as a cofactor to activate numerous cytoplasmic enzymes and participates in turgor adjustment, stomata movement, cell elongation, metabolism, growth, stress responses and other physiological processes (Gambale and Uozumi, 2006; Lebaudy *et al*., 2007; Marschner, 1995). Maintaining a high‐cytosolic K^+^:Na^+^ ratio is therefore critical for normal cellular function. However, elevated cellular Na^+^ content inhibits K^+^ absorption due to their similar physiochemical features that do not differentiate them from one another during cation‐transporting and enzyme reactions (Maathuis and Amtmann, 1999). Our analysis of K^+^ accumulation in both WT and TG creeping bentgrass revealed that the shoot potassium content in all TG plants was higher than or comparable to that in WT controls under normal conditions (Figure 4a). When subjected to salt stress, the shoot potassium contents of the TG lines harbouring only *OsSIZ1* or *Fld* either remained the same or declined compared to that under normal conditions (Figure 4a). Shoot potassium content of the WT controls and the other TG lines expressing either only *AVP1* or all three transgenes, *AVP1*, *OsSIZ1* and *Fld* increased significantly compared to that under normal conditions. It should be noted that except for the *OsSIZ1*‐expressing TG line, all the other TG plants and the WT controls still had similar shoot potassium accumulation under salt stress (Figure 4a). Considering the significantly lower Na^+^ accumulation in all the TG lines, especially in those expressing all three transgenes, *AVP1*, *OsSIZ1* and *Fld* (COE1‐3), a high K^+^:Na^+^ ratio in the TG lines, particularly in COE1‐3, when compared to WT controls was maintained, contributing to enhanced salinity tolerance. In roots, the potassium content in all TG plants was significantly higher than that in WT controls under normal conditions. When subjected to salt stress, the potassium accumulation in the TG lines expressing all three transgenes, *AVP1*, *OsSIZ1* and *Fld* remained the same as that under normal conditions, whereas the WT controls and the TG lines expressing only one of the three genes, *AVP1*, *OsSIZ1* or *Fld* exhibited a significantly lower potassium accumulation than the TG lines expressing all three transgenes, *AVP1*, *OsSIZ1* and *Fld* (Figure 4b). It should be noted that the root potassium accumulation in all three TG lines expressing one of the three transgenes, *AVP1*, *OsSIZ1* or *Fld* was still significantly higher than that in the WT controls (Figure 4b). Although the TG lines co‐expressing the three pyramided transgenes, *AVP1*, *OsSIZ1* and *Fld* (COE1‐3) had higher root Na^+^ accumulations than the WT controls under salinity stress (Figure 4a), the well‐maintained K^+^ accumulations in the COE1‐3 plants led to higher root K^+^:Na^+^ ratios than the WT controls as observed in the shoot (Figure 4a), contributing to enhanced salinity tolerance.

Calcium is an essential mineral involved in signal transduction. Its analysis in both TG and WT plants revealed that all TG lines had a significantly higher shoot and root calcium accumulation than WT controls under normal conditions (Figure 4a,b). When subjected to salt stress, the shoot calcium accumulation increased significantly in WT controls, but sharply declined in all TG plants, and as such, the shoot calcium content in WT controls was significantly higher than that in all TG lines (Figure 4a). Intriguingly, the root calcium content remained unchanged in WT controls but significantly declined in all TG plants under salt stress (Figure 4b). Even so, the WT calcium content was still significantly lower than that of all TG plants (Figure 4b).

### Co‐overexpression of *AVP1*, *OsSIZ1* and *Fld* improves plant drought tolerance associated with enhanced proline accumulation

**Figure 5:**
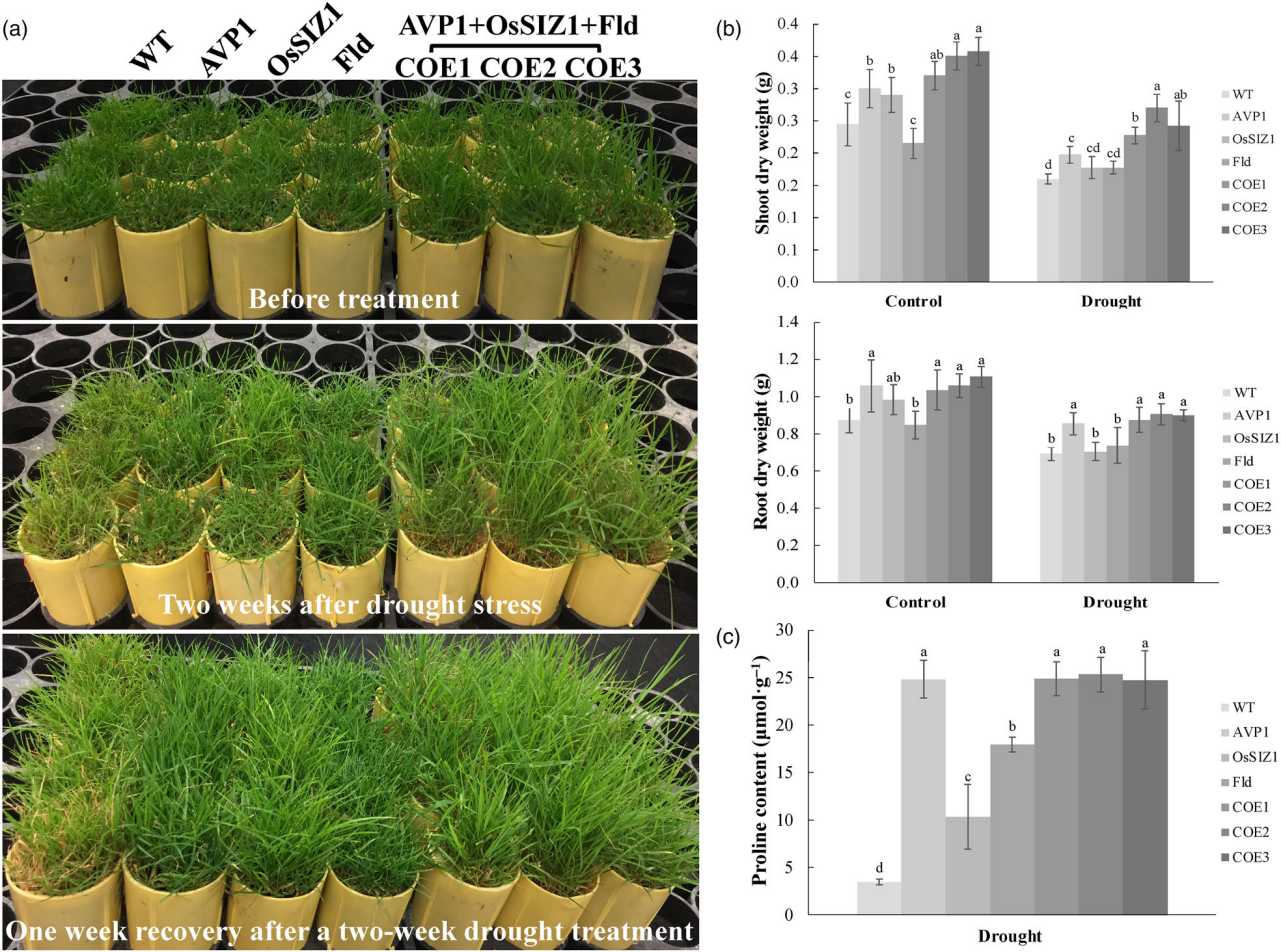
Plant performance under drought stress. (a) TG lines expressing all three transgenes, *AVP1, OsSIZ1* and *Fld* (TG1‐3) or only one of them, *AVP1* (Li *et al*., 2010), *OsSIZ1* (Li *et al*., 2013) or *Fld* (Li *et al*., 2017) as well as the non‐TG WT controls initiated from the same number of tillers were fully developed in cone‐tainers for 10 weeks under normal conditions in growth room. The 10 weeks old plants were then uniformly trimmed and grown for one more week (top panel) before being subjected to 2 weeks of limited water supply (middle panel). The bottom panel shows plants 1 week after recovery from a 2‐week drought stress. (b) Shoot and root DWs of the WT plants and various TG lines grown under normal conditions (control) or subjected to 2 weeks of water withholding. (c) Proline contents of the WT controls and various TG lines under drought stress for 2 weeks. Data are shown as means (*n* = 3); error bars represent standard deviation. Statistically significant differences between various plant lines were determined by one‐way ANOVA. Posthoc comparisons using Tukey's HSD test were conducted to determine the overall difference between groups. Means not sharing the same letter are statistically significantly different (*P* < 0.05).

To study how stacked expression of *AVP1*, *OsSIZ1* and *Fld* impacts plant drought response, we applied drought stress through 2 weeks of limited water supply to both the WT control plants and the TG lines harbouring all three transgenes or only one transgene. The stressed plants were then thoroughly watered for 2 weeks to allow recovery. We analysed plant performance and observed that drought stress significantly inhibited plant growth and led to a declined biomass production, more severe in shoots than in roots (Figure 5a,b). However, the TG plants expressing all three genes, *AVP1*, *OsSIZ1* and *Fld*, displayed significantly less growth inhibition with a higher shoot and root biomass than the WT controls, and the TG plants expressing only one of the three transgenes except *AVP1* TG plants, which had a similar root growth to the three genes‐expressing TG lines (Figure 5a,b). No significant difference was observed among WT or the TG plants expressing *OsSIZ1* or *Fld* (Figure 5a,b). Phenotypically, all TG lines outperformed the WT control plants in drought response. As shown in Figure 5a, all stressed TG plants remained largely turgid and green without apparent damage and rapidly recovered upon stress release, whereas the stressed WT controls displayed dehydration symptoms, such as loss of turgor and wilting and remained unhealthy with 30%–40% dead tillers 1 week after recovery (Figure 5a).

Proline, an osmoprotectant involved in lowering the solute potential of plant cells has been implicated in mediating plant response to various environmental adversities (Li *et al*., 2010; Nanjo *et al*., 1999a, 1999b; Yamada *et al*., 2005). To reveal whether stacked *AVP1*, *OsSIZ1* and *Fld* impacts plant proline production, we measured proline content in WT and TG lines under drought stress; all TG lines produced significantly more proline than the WT controls (Figure 5c). Proline accumulation was highest in the TG plants expressing all three genes, *AVP1*, *OsSIZ1* and *Fld*, and those expressing only *AVP1*, followed by the *Fld*, and then the *OsSIZ1* TG plants (Figure 5c).

### Co‐overexpression of *AVP1*, *OsSIZ1* and *Fld* enhances plant heat tolerance

**Figure 6:**
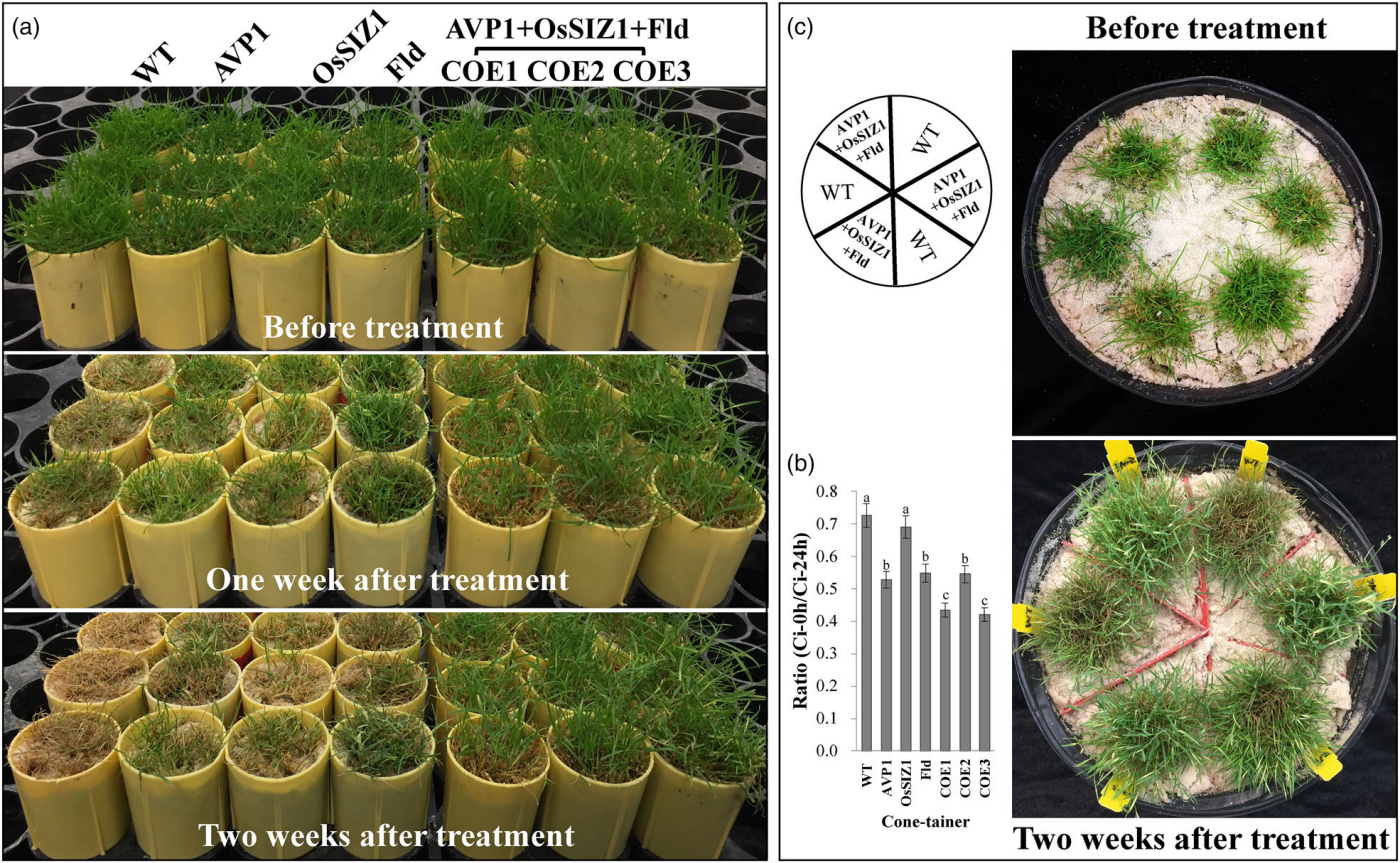
Plant performance under heat stress. (a) TG lines expressing all three transgenes, *AVP1, OsSIZ1* and *Fld* (TG1‐3) or only one of them, *AVP1* (Li *et al*., 2010), *OsSIZ1* (Li *et al*., 2013) or *Fld* (Li *et al*., 2017) as well as the non‐TG WT controls initiated from the same number of tillers were fully developed in cone‐tainers for 10 weeks under normal conditions in growth room. The 10 weeks old plants were then uniformly trimmed and grown for one more week (top panel) before being subjected to a heat stress initially at 35 °C in the light and 30 °C in the dark for 1 week (middle panel), and then at 40 °C in the light and 35 °C in the dark for one more week (bottom panel) with a relative humidity of 60%–80%. (b) Leaf electrolyte leakage (EL) of the WT controls and various TG lines under heat stress for 2 weeks. Data are shown as means (*n* = 3); error bars represent standard deviation. Statistically significant differences between various plant lines were determined by one‐way ANOVA. Posthoc comparisons using Tukey's HSD test were conducted to determine the overall difference between groups. Means not sharing the same letter are statistically significantly different (*P* < 0.05). (c) Performance of the TG plants expressing all three genes, *AVP1*, *OsSIZ1* and *Fld* and the WT controls under heat stress. Ten‐week‐old plant lines grown together in big pots (top) were subjected to heat stress at 40 °C in the light and 35 °C in the dark with a relative humidity of 85% for 2 weeks (bottom).

To reveal whether stacked *AVP1*, *OsSIZ1* and *Fld* affect plant thermotolerance, we applied heat stress to both the WT control plants and the TG lines harbouring all three transgenes or only one transgene for performance evaluation and found that all the plants tested exhibited heat‐inflicted symptoms, but with striking differences in severity (Figure 6a,c). The damage was most pronounced in the WT controls, followed by the TG lines expressing one of the three transgenes, *AVP1*, *OsSIZ1* or *Fld*, whereas the TG plants expressing all three transgenes showed the least severe symptoms (Figure 6a,c). When plants grown in the small cone‐tainers were subjected to 2 weeks of heat stress, nearly, all WT plants wilted and died. The TG lines expressing all three transgenes, *AVP1*, *OsSIZ1* and *Fld*, remained predominantly green and still grew normally (Figure 6a). The TG lines expressing only one of the three transgenes also survived the stress but suffered from significant heat‐inflicted damages (Figure 6a). The enhanced heat tolerance in TG plants, especially in those expressing all three transgenes, was further associated with an improved cell membrane integrity compared to the WT controls as the leaf cell EL accumulation in the TG plants expressing all three transgenes or the TGs expressing only *AVP1* or *Fld* was significantly less than in the WT controls (Figure 6b).

### TGs expressing *AVP1*, *OsSIZ1* and *Fld* exhibit an enhanced tolerance to N starvation associated with elevated N accumulation

**Figure 7:**
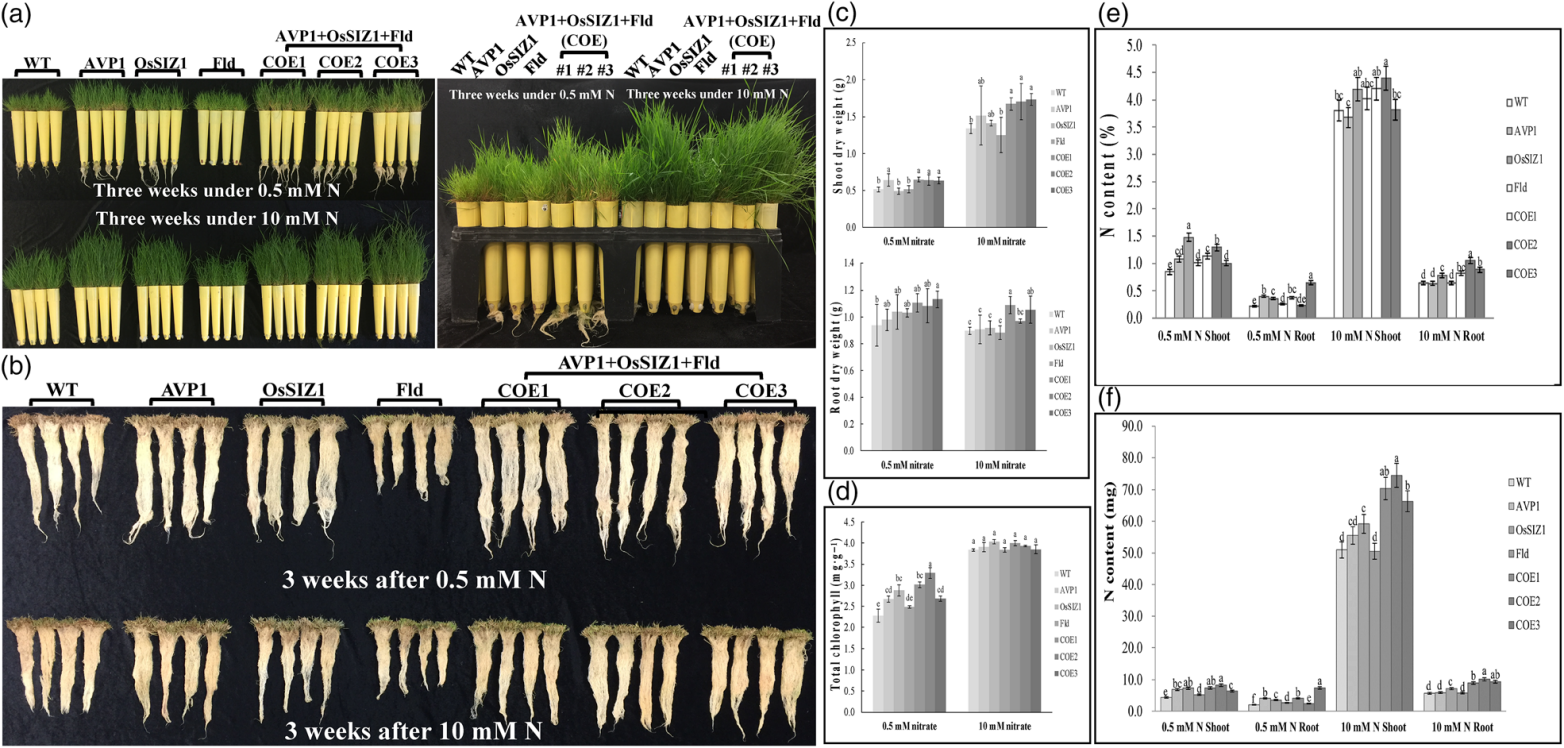
Plant performance under nitrogen (N) starvation. (a) Ten‐week‐old WT plants and different TG lines were uniformly trimmed, and then grown under N‐starved (0.5 mM N, left top and right panels) or N‐sufficient (10 mM N, left bottom and right panels) conditions for 3 weeks. (b) Plant root development under N‐starved (0.5 mM N, top panel) or N‐sufficient (10 mM N, bottom panel) conditions for 3 weeks. (c) Shoot and root DWs of the WT plants and various TG lines grown under N‐starved (0.5 mM N) or N‐sufficient (10 mM N) conditions for 3 weeks. (d) Total chlorophyll contents of the WT plants and various TG lines grown under N‐starved (0.5 mM N) or N‐sufficient (10 mM N, bottom panel) conditions for 3 weeks. (e) Percentages of shoot and root total N contents of the WT plants and various TG lines measured 3 weeks after N starvation (0.5 mM N) or growing under N‐sufficient (10 mM N) conditions. (f) Weights of shoot and root total N contents of the WT plants and various TG lines measured 3 weeks after N starvation (0.5 mM N) or growing under N‐sufficient (10 mM N) conditions. Data are shown as means (*n* = 3); error bars represent standard deviation. Statistically significant differences between various plant lines were determined by one‐way ANOVA. Posthoc comparisons using Tukey's HSD test were conducted to determine the overall difference between groups. Means not sharing the same letter are statistically significantly different (*P* < 0.05).

Nitrogen (N) plays an important role in shaping overall plant growth and development. Monocot plants like creeping bentgrass are even more sensitive to N deficiency than dicots. We have previously demonstrated that Fld mediates N assimilation and metabolism, and creeping bentgrass overexpressing *Fld* exhibits significantly increased N accumulation and total chlorophyll content. (Li *et al*., 2017; Zurbriggen *et al*., 2008). This prompted us to investigate whether Fld and the other two stress‐related proteins, AVP1 and OsSIZ1 would synergistically impact plant performance under N starvation. To this end, we grew WT controls and TG lines under two previously established regimes of N application, N‐starved (0.5 mM nitrate) and N‐sufficient (10 mM nitrate) conditions (Li *et al*., 2016; Yuan *et al*., 2015), and compared plant responses. As shown in Figure 7a–c, although N starvation severely inhibited plant growth, the TG lines expressing all three transgenes, *AVP1*, *OsSIZ1* and *Fld*, and those expressing only *AVP1* grew much more vigorously than WT controls and the other two TG lines expressing *OsSIZ1* or *Fld*, exhibiting significantly enhanced shoot biomass production under both N‐starved and sufficient conditions. Similar performance was also observed in root growth. The TG lines expressing all three transgenes, *AVP1*, *OsSIZ1* and *Fld*, developed a significantly more robust root system with a higher root biomass than WT controls and the other three TG lines expressing only *AVP1*, *OsSIZ1* or *Fld* under sufficient N supply (Figure 7b,c). Intriguingly, although N starvation severely inhibited plant shoot development, it significantly stimulated root growth in all plants tested, leading to more vigorously grown, longer roots. The stimulation was more pronounced in the TG lines than in the WT controls (Figure 7a–c). Overall, the TG plants expressing all three transgenes, *AVP1*, *OsSIZ1* and *Fld*, produced a significantly greater biomass under both sufficient and N starvation conditions than the WT controls and the other three TG lines expressing *AVP1*, *OsSIZ1* or *Fld*. Of the different TG lines, the total biomass of the TG plants expressing all three transgenes, *AVP1*, *OsSIZ1* and *Fld* was the highest, whereas that of the TG line expressing only *Fld* was the least.

Further analysis of plant leaf total chlorophyll contents revealed no significant difference among all plant lines tested under N‐sufficient conditions (Figure 7d). However, N starvation significantly reduced leaf chlorophyll contents, which was more pronounced in the WT controls than in all the TG lines (Figure 7d). This suggests that TG plants may be less prone to N starvation‐inflicted chlorophyll degradation and therefore maintain a higher capacity for photosynthesis than the WT controls under N starved conditions. This is consistent with their greater shoot and root DWs compared to WT controls after 3 weeks of 0.5 mM N treatment (Figure 7a–c).

To establish a direct link between N accumulation and plant growth and understand how transgene expression would impact plant N uptake, we measured the total shoot and root N contents in both WT and TG lines under N‐starved and sufficient conditions. As shown in Figure 7e, N starvation led to a significant decline in N uptake in both WT and TG plants compared to the N‐sufficient condition. However, a generally higher plant N accumulation, especially in the shoot, in all TG lines in comparison to the WT controls was observed under both N‐sufficient and starved conditions (Figure 7e). Considering the significantly higher shoot and root biomass production in the TG plants than in WT controls under both conditions, the enhanced total N uptake in the TG plants was even more pronounced compared to the WT controls, indicating an enhanced N utilization efficiency (NUE) in TG plants, especially those co‐expressing all three genes, *AVP1*, *OsSIZ1* an *Fld* (Figure 7f).

### Co‐overexpression of *AVP1*, *OsSIZ1* and *Fld* leads to enhanced plant performance under phosphate starvation conditions

**Figure 8:**
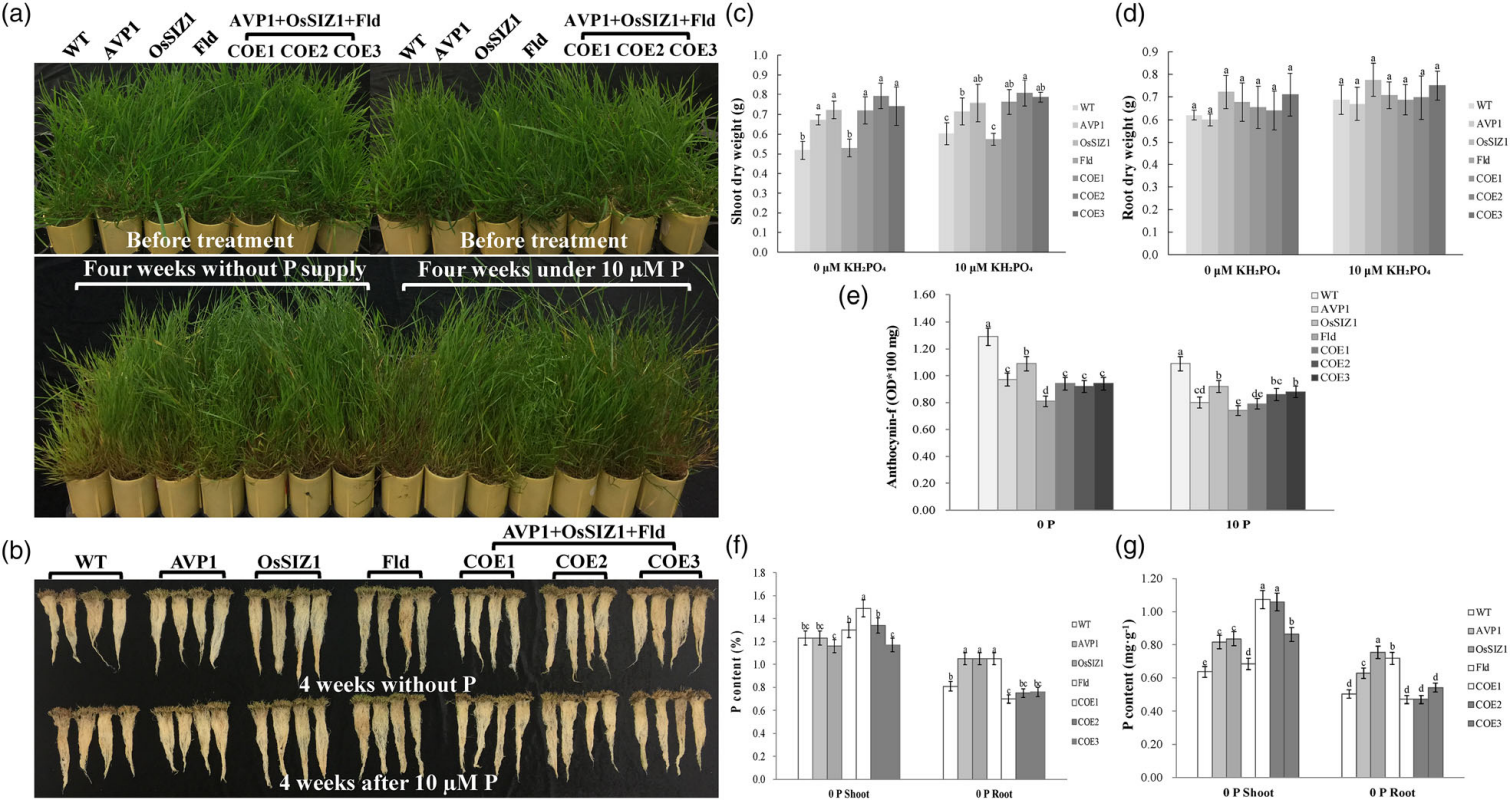
Plant performance under phosphate (Pi) starvation. (a) Ten‐week‐old WT plants and different TG lines (top panel) were uniformly trimmed and grown for two more weeks before subjecting to Pi starvation (0 μM or 10 μM Pi) for 4 weeks (bottom pane). (b) Plant root development under Pi starvation (0 μM or 10 μM Pi) for 4 weeks. (c) Shoot DWs of the WT plants and various TG lines grown under Pi starvation (0 μM or 10 μM Pi) for 4 weeks. (d) Root DWs of the WT plants and various TG lines grown under Pi starvation (0 μM or 10 μM Pi) for 4 weeks. (e) Anthocyanin contents of the WT plants and various TG lines grown under Pi starvation (0 μM or 10 μM Pi) for 4 weeks. (f) Percentages of shoot and root total P contents of the WT plants and various TG lines measured 4 weeks after Pi starvation (0 μM or 10 μM Pi) (g) Weights of shoot and root total P contents of the WT plants and various TG lines measured 4 weeks after Pi starvation (0 μM or 10 μM Pi). Data are shown as means (*n* = 3); error bars represent standard deviation. Statistically significant differences between various plant lines were determined by one‐way ANOVA. Posthoc comparisons using Tukey's HSD test were conducted to determine the overall difference between groups. Means not sharing the same letter are statistically significantly different (*P* < 0.05).

SIZ1‐mediated sumoylation is part of the signalling pathway implicated in coordinating plant responses to Pi starvation (Miura *et al*., 2005). Overexpression of a rice *SIZ1* gene, *OsSIZ1* in creeping bentgrass helps plants cope with Pi deficiency (Li *et al*., 2013), leading to a significantly less pronounced Pi starvation‐inflicted growth inhibition and anthocyanin accumulation in the *OsSIZ1* TG plants than in the non‐TG controls. Given the multiple roles AVP1 and Fld play in determining plant development and stress responses (Li *et al*., 2010, 2016), we were curious about their possible involvement in regulating plant Pi homeostasis and, if so, how the stacking of all three genes, *OsSIZ1*, *AVP1* and *Fld* would impact overall plant performance under Pi‐stared condition. To investigate this, we compared the growth of WT and all TG plants under Pi starvation conditions for 4 weeks. We observed that all plants tested became purple and chlorotic (Figure 8a), a symptom typical of Pi deficiency due to an increased accumulation of anthocyanin, which was significantly less in the TG lines than in the WT controls (Figure 8e). This suggests a better adaptation of the TG plants to the Pi‐stressed condition than the WT controls. Pi starvation also inflicted growth inhibition in all plant lines tested. However, the TG plants expressing all three transgenes, *AVP1*, *OsSIZ1* and *Fld*, produced significantly more shoot biomass (44.23%) than WT controls in the absence of Pi (0 μM KH_2_PO_4_). When Pi supply increased to 10 μM, plant growth improved but the TG plants expressing all three transgenes, *AVP1*, *OsSIZ1* and *Fld*, still produced 31.67% more shoot biomass than the WT controls (Figure 8a,c). The differences among TG plants were mostly insignificant except for the TG line expressing only *Fld*, whose shoot DW was comparable to or lower than that of the WT controls under Pi starvation (Figure 8a,c). On the contrary, the root DW did not show any significant differences among the tested plants under both treatments of Pi starvation. However, most of the TG lines, especially those expressing all three transgenes, showed a tendency for higher root DW than the WT controls (Figure 8b,d). Of particular note, although the shoot DW of the TG plants expressing only *Fld* was much lower than that of the other TG plants under Pi starvation (Figure 8a,c), its root biomass was not significantly different from the other TG lines (Figure 8b,d). Hence, the Pi starvation‐inflicted root growth inhibition in the TG plants expressing only *Fld* was less pronounced than in the other plant lines tested, giving rise to a root length (Figure 8b) and a root DW comparable to that of the other TG lines tested (Figure 8d). This is in contrast with their shorter root phenotype than the other TG lines observed when grown under normal conditions (Figures 2d and 3a).

### TGs harbouring *AVP1*, *OsSIZ1* and *Fld* display differential expression patterns in other representative genes implicated in plant development and abiotic stresses responses

**Figure 9:**
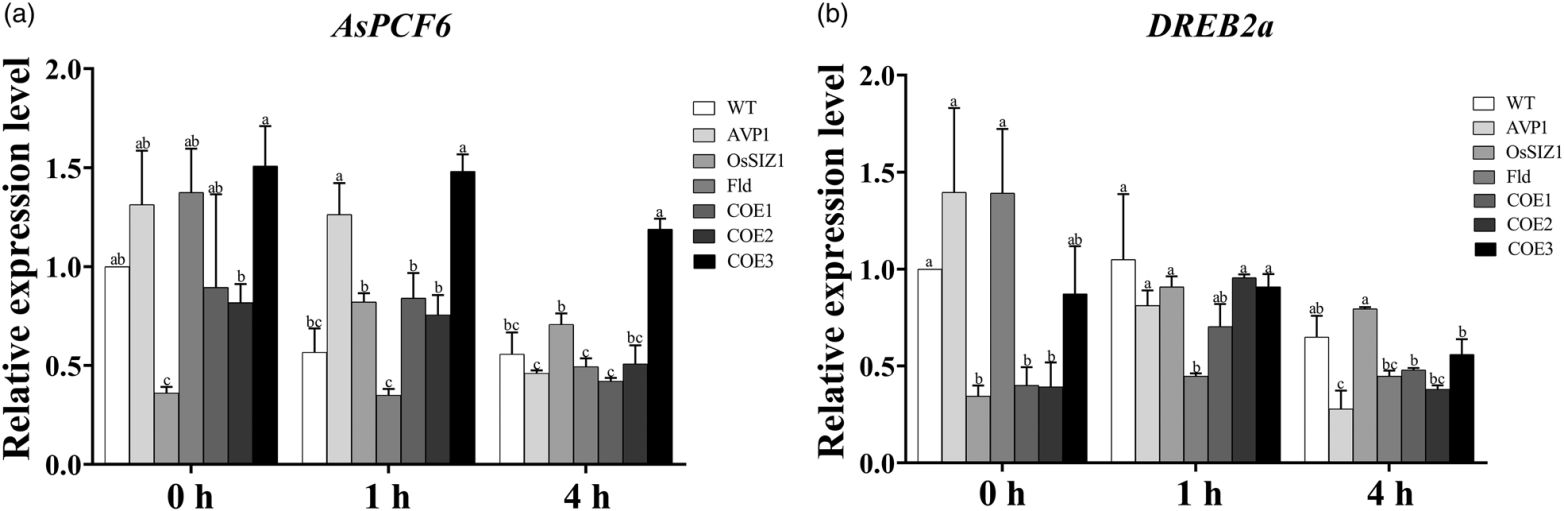
Expression of development‐ and stress‐responsive genes in WT and TG creeping bentgrass. Expression of *AsPCF6* (a) and *DREB2a* (b) in plants subjected to 150 mM NaCl treatment for 0, 1 and 4 h. Total RNA was extracted from 1 g of young leaves of TG lines expressing all three genes, *AVP1, OsSIZ1* and *Fld* (TG1‐3) or only one of them, *AVP1* (Li *et al*., 2010), *OsSIZ1* (Li *et al*., 2013) or *Fld* (Li *et al*., 2017) as well as WT controls. Gene expression was determined by RT–PCR on cDNA to amplify *AsPCF6* and *DREB2a*. The ΔΔCt method was used to analyse the relative gene expression levels. *AsACT1* was used as the internal control. Data are presented as means of three biological replicates × three technical replicates; error bars represent standard deviation. Statistically significant differences between various plant lines were determined by one‐way ANOVA. Posthoc comparisons using Tukey's HSD test were conducted to determine the overall difference between groups. Means not sharing the same letter are statistically significantly different (*P <* 0.05).

To gain an insight into the underlying mechanism of how the co‐expression of *AVP1*, *OsSIZ1* and *Fld* improves plant development and stress resistance, we investigated *AsPCF6* (Zhou *et al*., 2013) and *AsDREB2a* (Liu *et al*., 1998; Sakuma *et al*., 2006a,b), the two representative genes encoding different transcription factors previously implicated in plant development and response to various abiotic stresses. Their expression was analysed in WT and various TG lines under normal and salinity conditions. As shown in Figure 9a, under normal condition, all the TG lines except the *OsSIZ1*‐expressing TGs had either similar *AsPCF6* expression to the WT controls (COE1 and COE2) or exhibited a tendency of higher, but not significantly, *AsPCF6* expression than the WT controls (*AVP1*‐expressing TG line and COE3). The *OsSIZ1*‐expressing TG plants exhibited significantly lower *AsPCF6* expression than the WT controls. Upon salt stress, *AsPCF6* expression decreased in all plant lines tested. However, the decline in *AsPCF6* expression occurred 1 h after salt treatment in the WT controls, a process that occurred much more rapidly than in the three TG lines co‐expressing *AVP1*, *OsSIZ1* and *Fld* (COE1‐3) and the *AVP1*‐expressing TG plants, which did not take place until 4 h after salinity stress. Interestingly, *AsPCF6* expression in the *OsSIZ1*‐expressing TG plants was significantly induced upon salt stress and remained elevated even 4 h after salt treatment, whereas that in the *Fld*‐expressing TG line declined significantly upon salt stress.

*DREB2a* expression in the WT controls, *AVP1*‐ and *Fld*‐expressing TG lines and one of the three TG lines co‐expressing *AVP1*, *OsSIZ1* and *Fld* (COE3) was significantly higher than that in the *OsSIZ1*‐expressing plants and two of the three TG lines co‐expressing *AVP1*, *OsSIZ1* and *Fld* (COE1 and COE2) under normal condition (Figure 9b). However, 1 h after salinity stress, *DREB2a* expression in the *OsSIZ1*‐expressing and the COE1 and COE2 TG lines was significantly elevated, reaching a similar level to that in the WT controls, *AVP1*‐ and *Fld*‐expressing and COE3 TG lines (Figure 9b). Intriguingly, prolonged salt stress (4 h) led to declined *DREB2a* expression in all the plant lines tested, which was more pronounced in the *AVP1*‐expressing TG line and those co‐expressing *AVP1*, *OsSIZ1* and *Fld* (COE1, COE2 and COE3) (Figure 9b).

### Comparative transcriptomic profiling between WT and the TG creeping bentgrass expressing *AVP1*, *OsSIZ1* and *Fld*

**Figure 10:**
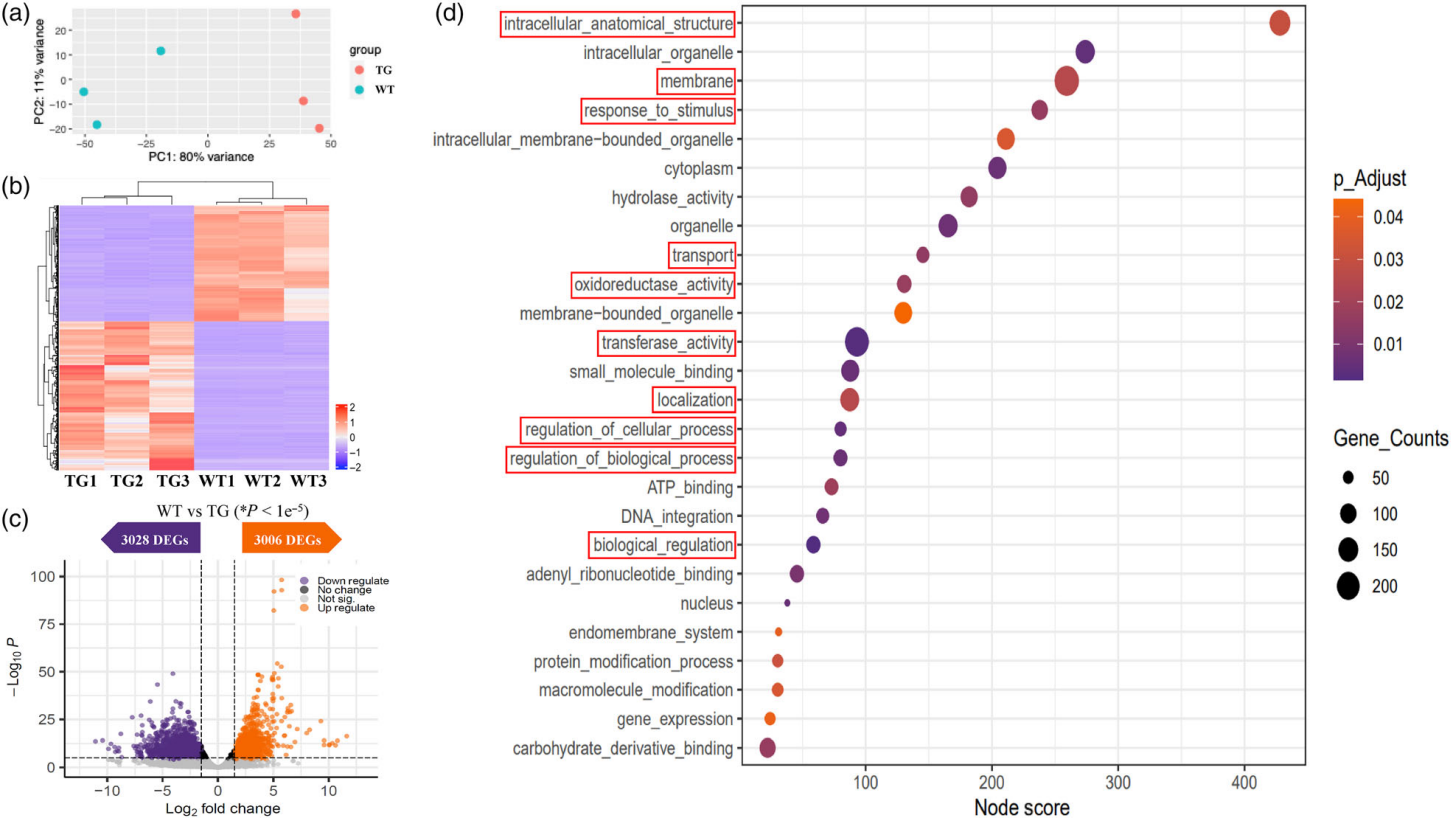
Global transcription analysis of gene expression in WT and the TG plants co‐expressing *AVP1*, *OsSIZ1* and *Fld*. (a) Principal component (PC) analysis testing reproducibility of RNA‐seq analysis. The plot shows the expected consistency between three biological replicates of WT and the TG samples, respectively. (b) Heatmap illustrating fold change of genes differentially expressed in the TG versus WT control plants gated at *P*‐value < 1e^−7^. (c) Volcano plot of gene transcript abundance in the TG versus WT as Log_2_‐fold changes (FC) versus −Log_10_(*P*) at false discovery rate (FDR) adjusted *P*‐value < 1e^−5^. Among the differentially expressed transcripts (Log_2_ FC > 1.5 or < −1.5), 3006 are up‐regulated (orange) and 3028 are down‐regulated (blue) in the TG plants versus WT controls. (d) Gene Ontology (GO) enrichment analysis of the annotated differentially expressed genes (DEGs) in the TG and WT plants. Shown in the graphic are identified GO terms with the enrichment FDR adjusted *P*‐value <0.05, number of genes per GO term, and node score for each GO term (http://bfcd.blast2go.com/user/manual/; Conesa *et al*., 2005). Each bubble represents a GO term whose size and colour represent the number of genes and significance of the enrichment, respectively. The identified GO terms are grouped into three GO categories including biological process, molecular function and cellular component. Development‐ and stress response‐related GO terms are high‐lighted in red rectangles.

To further elucidate key molecular pathways governing improved plant growth and significantly enhanced stress resistance via the synergistic coordination of the three co‐expressed transgenes, *AVP1*, *OsSIZ1* and *Fld*, we conducted whole‐genome gene expression analysis using RNA sequencing to study the differentially expressed genes (DEGs) in WT and the TG lines harbouring *AVP1*, *OsSIZ1* and *Fld*. A volcano plot shows the distribution of Log_2_‐fold changes (FC) of TG compared with WT data sets at false discovery rate (FDR) adjusted *P*‐value < 0.05. Among the differentially expressed transcripts (Log_2_ FC > 1.5 or <−1.5, adjusted *P* < 1e^−5^), 3006 are up‐regulated and 3028 down‐regulated in the TG plants compared with WT controls (Figure 10c). Functional annotation of putative gene products revealed that co‐expression of *AVP1*, *OsSIZ1* and *Fld* impacted multiple biological processes including intracellular anatomic structure, response to stimulus, membrane, transport, oxidoreductase activity, regulation of cellular process, regulation of biological process, biological regulation (Figure 10d). Based on the properties and functions of *AVP1*, *OsSIZ1* and *Fld*, and the significant altered plant phenotypes in TGs co‐expressing these genes, we focused on DEGs involved in plant development and stress responses. We observed that of the top 1000 genes differentially regulated with statistically highest significance (*P* < 0.05) between WT and the TGs co‐expressing *AVP1*, *OsSIZ1* and *Fld*, approximately 15% of them encode proteins involved in regulating plant development and stress responses, including receptor such as protein kinase, thioredoxin, glutaredoxin, peroxidase, catalase and glutathione transferase, aquaporin, osmotin, ABC transporter, ion, amino acid and polyol transporters, pyrophosphate‐energized vacuolar membrane proton pump, calcium sensor, development‐ and stress response‐related transcription factors, auxin, gibberellin and ethylene signalling, expansin‐like proteins, ubiqitination and chaperone proteins as well as disease resistance proteins, methyltransferase and demethylase (Table 1).

**Table 1:** Nonexhaustive list of plant development and/or stress response‐related candidates from the top 1000 genes differentially regulated with statistically highest significance (*P* < 0.05) between wild type (WT) and the TGs co‐expressing *AVP1*, *OsSIZ1* and *Fld*

Annotated gene function	Number
Receptor like protein kinase	39
Thioredoxin, glutaredoxin, peroxidase, catalase and glutathione transferase	25
Aquaporin, osmotin, ABC transporter, ion, amino acid and polyol transporters	36
Disease resistance proteins	18
Pyrophosphate‐energized vacuolar membrane proton pump	3
Calcium sensor	5
Development‐ and stress response‐related transcription factors	35
Auxin, gibberellin and ethylene signalling	12
Expansin‐like proteins	6
Ubiquitination, chaperone proteins	6
Methyltransferase and demethylase	9

## Discussion

The introduction of multiple beneficial genes or alleles into new cultivars for their synergistic expression that enhances plant biotic and abiotic stress resistance and crop yield potential is one of the major goals in modern agricultural practice for enabling sustainable crop production (Dormatey *et al*., 2020; Lu *et al*., 2017; Munns, 2005). In this study, we have generated TG creeping bentgrass that simultaneously expresses *AVP1*, *OsSIZ1* and *Fld*, the three genes previously demonstrated to be involved in plant development and stress responses. We observed that TG plants co‐expressing the three transgenes performed exceptionally well compared with WT controls and the TGs overexpressing individual genes under both normal and various abiotic stress conditions, exhibiting significantly enhanced plant growth with elevated biomass production and highly improved plant tolerance to multiple abiotic stresses and various nutritional deficiencies including drought, salinity, heat and nitrogen and phosphate starvation. These results suggest that stacked *AVP1*, *OsSIZ1* and *Fld* genes function synergistically to regulate plant development and plant stress responses.

AVP1, OsSIZ1 and Fld have all been demonstrated to impact plant growth and development when individually manipulated in TGs. We previously showed that compared to the WT controls, constitutive expression of *AVP1* in TG creeping bentgrass led to significantly enhanced shoot and root biomass production under both normal and high‐salinity conditions (Li *et al*., 2010), consistent with the observations in *Arabidopsis*, barley; tomato, corn, cotton and lettuce (Asad *et al*., 2008; Li *et al*., 2005, 2008; Lv *et al*., 2008, 2009; Paez‐Valencia *et al*., 2013; Park *et al*., 2005; Schilling *et al*., 2014, 2017). Similarly, overexpression of *OsSIZ1* in TG creeping bentgrass enhanced plant photosynthesis and shoot biomass production compared to the WT controls (Li *et al*., 2013). On the contrary, *Arabidopsis* and rice *OsSIZ1* mutants displayed a reduction in leaf size, plant height and biomass production (Catala *et al*., 2007; Miura *et al*., 2010; Park *et al*., 2010; Wang *et al*., 2011). AVP1‐ and OsSIZ1‐mediated enhancement of plant growth has been postulated to be associated with altered hormone biosynthesis and signalling as well as light signalling and chlorophyll biosynthesis. For example, SIZ1 has been demonstrated to regulate brassinosteroid, auxin and salicylic acid biosynthetic and signalling pathways, impacting the expression of the cell wall loosening‐ and cell expansion‐related genes and consequently, cell growth and plant development (Catala *et al*., 2007; Li *et al*., 2013; Miura *et al*., 2010). We have also previously shown that AVP1 regulates auxin transport from shoot to root, promoting robust root growth in TG creeping bentgrass (Li *et al*., 2010).

Unlike *AVP1* and *OsSIZ1*, Fld negatively impacts plant growth. Overexpression of *Fld* resulted in reduced biomass production and a modified inflorescence in TG creeping bentgrass (Li *et al*., 2017). Similar phenotypes were also observed in TG tomato in which expression of the cyanobacterial *Fld* led to a significant size reduction in stems, leaves and fruits despite an increased harvest index resulting from the production of higher fruit number per plant in smaller plants (Mayta *et al*., 2019). The negative impact on plant growth by the small electron transfer protein, Fld was presumably due to a potential modification in how the reducing equivalents generated in the photosynthetic electron transport chain (PETC) are distributed to the critical regulatory and metabolic pathways related to plant development (Li *et al*., 2017). Alternatively, *Fld* overexpression may alter cell ROS homeostasis and therefore, related ROS‐mediated signalling pathways, negatively impacting plant growth (Chan *et al*., 2016; D'Autreaux and Toledano, 2007; Dietz *et al*., 2015, 2016; Li *et al*., 2017; Mittler *et al*., 2011).

In this study, the pronounced positive effects of either AVP1 or OsSIZ1 on plant growth were even more significantly boosted to a new level when co‐expressed in TG creeping bentgrass, consistent with the observations in *Arabidopsis* and cotton (Esmaeili *et al*., 2019, 2021), whereas simultaneous *Fld* expression did not compromise the AVP1‐ and OsSIZ1‐mediated promotion in plant growth. Since AVP1 and OsSIZ1 participate in the biosynthesis and signalling of multiple hormones as well as chlorophyll biosynthesis and light signalling, simultaneous manipulation of these two genes in TGs most likely facilitates a coordinated crosstalk of various regulatory and metabolic pathways implicated in plant development, resulting in a significant boost in plant growth. The synergistic effects of AVP1 and OsSIZ1 may also offset the Fld‐triggered negative impact on plant growth.

AVP1, OsSIZ1 and Fld have also been implicated in regulating plant responses to various abiotic stresses (Dametto *et al*., 2015; Juan *et al*., 2014; Schilling *et al*., 2017) as revealed in our previous work in TG creeping bentgrass, in which *AVP1* overexpression resulted in significantly improved plant salt tolerance associated with enhanced capacity in water retention, proline accumulation and sodium sequestration into vacuoles (Li *et al*., 2010). In addition, constitutive expression of *OsSIZ1* led to enhanced drought and heat tolerance, elevated phosphate utilization efficiency and potassium uptake with a robust root system, and improved water retention and cell membrane integrity (Li *et al*., 2013). A further attempt to improve crop stress response by taking advantage of candidate genes from heterologous species led us to introduce the cyanobacterial *Fld* into creeping bentgrass. We observed that Fld TGs displayed drastically enhanced tolerance to various environmental adversities including oxidative, drought and heat stresses as well as nitrogen starvation associated with improved water retention, cell membrane integrity and altered expression of heat‐shock protein genes and up‐regulation of nitrite reductase and nitrogen transporter genes (Li *et al*., 2017). These results demonstrated the beneficial effects of each one of the three transgenes, *AVP1*, *OsSIZ1* and *Fld*, in plant adaption to various resilient environments, which prompted us to examine how they would work together to regulate plant stress responses, impacting plant performance under multiple environmental adversities. In this study, when *AVP1*, *OsSIZ1* and *Fld* were all constitutively expressed in creeping bentgrass, TGs harbouring the three genes outperformed the WT controls, and TG lines overexpressing only one individual transgene under various abiotic stress conditions, exhibiting significantly enhanced tolerance to salinity (Figure 3), drought (Figure 5) and heat stresses (Figure 6) as well as nitrogen (Figure 7) and phosphate starvation (Figure 8) accompanied by various altered physiological and biochemical characteristics. These observations indicate that the TG plants harbouring *AVP1*, *OsSIZ1* and *Fld* combine multiple agronomically beneficial traits brought by each one of them, exhibiting a superior overall plant performance under various environmental adversities. The results suggest that when co‐expressed in TGs, *AVP1 OsSIZ1* and *Fld* function synergistically to positively regulate plant stress responses.

In an effort of exploring molecular mechanisms of AVP1/OsSIZ1/Fld‐mediated plant growth and stress responses, we analysed two transcription factor genes, *AsPCF6* and *AsDREB2a* in WT and the TG plants. PCFs, the plant‐specific transcription factors belong to TCP (TEOSINTE BRANCHED/CYCLOIDEA/PROLIFERATING CELL FACTOR [PCF]) protein family that shares a conserved TCP domain with a basic helix–loop–helix structure. The TCP family is involved in plant development, such as leaf morphogenesis, by regulating cell proliferation (Nag *et al*., 2009; Ori *et al*., 2007; Palatnik *et al*., 2003). The expression of *AsPCF6* and several other *PCF* genes is also regulated by salt and drought stress (Zhou *et al*., 2013). The differential expression patterns of *AsPCF6* in the TG lines, especially in those harbouring the three transgenes, *AVP1*, *OsSIZ1* and *Fld*, in response to environmental cues, such as salt stress as observed in this study (Figure 9a) due to the synergistic effect conferred by the co‐expression of *AVP1*, *OsSIZ1* and *Fld*, may cause differential rather than normal signalling, altering related regulatory networks to affect the expression of the genes involved in various aspects of plant development and stress responses, and therefore resulting in elevated plant biomass production and significantly improved plant performance under varied environmental abiotic stresses.

The active transcription factor DREB2a interacts with the cis‐acting dehydration‐responsive element (DRE) sequence of the target genes involved in drought, salt and heat stress responses to activate their expression (Liu *et al*., 1998; Qin *et al*., 2008; Sakuma *et al*., 2006a,b). *DREB2a* transcription is activated by dehydration and high‐salt stress and the induced DREB2a then transactivates DRE‐dependent gene expression to mitigate environmental adversities (Liu *et al*., 1998). This indicates the important role DREB2a plays in regulating plant stress responses. However, overexpression of *DREB2a* caused growth retardation of the TG plants under normal non‐stressed conditions (Liu *et al*., 1998), suggesting that DREB2a‐triggered overproduction of stress‐related proteins is likely to negatively impact plant development under normal conditions, causing retarded plant growth. In our study, we observed a lower *DREB2a* expression under normal conditions but a more rapid induction upon stress, and a more pronounced, quick decline thereafter in the *OsSIZ1*‐expressing plants and the TG lines co‐expressing *AVP1*, *OsSIZ1* and *Fld* than in the WT controls and the TG lines expressing *AVP1* or *Fld* (Figure 9b). This may reflect a delicately fine‐tuned and cost‐effective regulatory mechanism for stress response, which is mediated by the synergistic effect of AVP1, OsSIZ1 and Fld (Figure 9b). This mechanism helps maintain a low‐*DREB2a* expression under normal conditions for regular or enhanced plant growth but triggers a rapid *DREB2a* induction upon stresses to activate downstream stress‐responsive pathways to mitigate the environmental adversities, and then quickly resume normally low‐*DREB2a* expression (Figure 9b).

Comparative transcriptomic profiling between WT and the TG creeping bentgrass revealed that co‐expression of *AVP1*, *OsSIZ1* and *Fld* leads to differential expression in more than 6000 genes, many of which encode proteins involved in regulating plant development and stress responses (Figure 10, Table 1). Intriguingly, many disease resistance protein genes were differentially regulated between WT and the TG lines. While this may indicate a direct implication of disease resistance genes in plant abiotic stress responses, it may also imply a potential role AVP1, OsSIZ1 and Fld may play in regulating disease‐related genes to modulate plant biotic stress responses. The possible involvement of AVP1, OsSIZ1 and Fld in, or synergistic effect of their co‐expression on plant biotic stress responses is worth further investigation. Of note, the TG plants also exhibited an altered expression in nine methyltransferase and demethylase genes (Table 1), suggesting that AVP1, OsSIZ1 and Fld may play a role in modulating genome chromatin structure via DNA methylation, histone and/or posttranscriptional RNA modifications, and therefore, participate in epigenetic control of plant growth and stress responses.

In summary, co‐expression of *AVP1*, *OsSIZ1* and *Fld* synergistically impacts plant growth and plant responses to multiple environmental adversities including drought, salinity, hea and nutrition starvation stresses potentially through delicately fine‐tuned genetic and/or epigenetic regulation of the relevant development‐ and stress‐responsive genes. The data obtained from the current study not only highlight the importance of combined use of *AVP1*, *OsSIZ1* and *Fld* in producing TG crops with superior overall performance under both normal and adverse environments, but also provide new insights into gene stacking as an effective approach for plant genetic engineering. A similar strategy can be extended to the use of other beneficial genes in various crop species for trait modifications, enhancing agricultural production.

## Experimental procedures

### Plasmid construction and bacterial strains

The binary vector pSB11 (Hiei *et al*., 1994) was used to prepare the *AVP1*, *OsSIZ1* and *Fld* chimeric gene expression construct, p35S‐AVP1/35S‐OsSIZ1/35S‐Fld/35S‐bar (Figure 1a). The three genes, *AVP1*, *OsSIZ1* and *Fld* are all under the control of the cauliflower mosaic virus 35S (CaMV 35S) gene promoter and linked to *bar*, a selectable marker gene for herbicide resistance, also driven by the CaMV 35S promoter. The plasmid was generated by first cloning the *AVP1* expression cassette released from p35S‐AVP1 (Luo, unpublished) after *Xho*I digestion into the corresponding site of p35S‐bar (Luo, unpublished) to produce p35S‐AVP1/35S‐bar. The *Fld* expression cassette that contains the CaMV35S promoter‐driven coding sequence of a pea FNR chloroplast‐targeting transit signal peptide (Li *et al*., 2017) translationally fused to the cyanobacterial *Fld* gene was then released from p35S‐Fld (Luo, unpublished) by *Age*I digestion and cloned into the corresponding site of p35S‐AVP1/35S‐bar to produce p35S‐AVP1/35S‐Fld/35S‐bar. Finally, the *OsSIZ1* expression cassette released from p35S‐OsSIZ1 (Luo, unpublished) by *Avr*II digestion was inserted into the corresponding site of p35S‐AVP1/35S‐Fld/35S‐bar to produce p35S‐AVP1/35S‐OsSIZ1/35S‐Fld/35S‐bar (Figure 1a).

The final construct p35S‐AVP1/35S‐OsSIZ1/35S‐Fld/35S‐bar (Figure 1a) was eventually transferred into *Agrobacterium tumefaciens* strain LBA4404 by electroporation for use in creeping bentgrass transformation.

### Plant materials and transformation

Plant transformation in creeping bentgrass (*A. stolonifera* L.) cv. Penn A‐4 (supplied by PureSeed, Canby, OR) by *Agrobacterium* infection of embryogenic callus initiated from mature seeds was conducted essentially as previously described (Luo *et al*., 2004).

All the regenerated TG plants were transferred in commercial potting mixture soil (Fafard 3‐B Mix, Fafard Inc., Anderson, SC), and maintained in the greenhouse under a 16 h photoperiod with supplemental lighting at 27 °C in the light and 25 °C in the dark.

The previously generated TG lines expressing only *AVP1* (Li *et al*., 2010), *OsSIZ1* (Li *et al*., 2013) or *Fld* (Li *et al*., 2017) as well as non‐TG WT plants were used as controls to compare with the TG lines expressing all three genes, *AVP1*, *OsSIZ1* and *Fld*.

### Plant DNA, RNA isolation and gene expression analysis

Plant genomic DNA was isolated from 1 g of young leaves using the cetyltrimethylammonium bromide (CTAB) method (Luo *et al*., 1995) and used for PCR amplification of *bar* gene to screen for putative TG plants. The primers used for *bar* amplification are listed in Table S1.

Plant total RNA extracted with Trizol reagent (Invitrogen, Carlsbad, CA) was used for cDNA synthesis by M‐MuLV Reverse Transcriptase (New England Biolabs). *AsActin* was used as the internal reference gene for assessing gene expression levels in creeping bentgrass. Semi‐quantitative RT–PCR was conducted in a 25 μL system solution containing 1 μL Taq, 2.5 μL 10× buffer, 1 μL cDNA, 1 μL 10 mM dNTPs and 1 μL 10 μM primer F + R using the following program: denature at 95 °C for 3 min, followed by 30 cycles of 95 °C for 30 s, 60 °C for 30 s and 72 °C for 30 s, and then an extension step at 72 °C for 1 min. RT–PCR was conducted in a 20 μL system solution containing 10 μL Luna Universal qPCR Master Mix (New England Biolabs), 1 μL cDNA, 2 μL 2 μM primer F + R using the following program: denature at 95 °C for 3 min, followed by 40 cycles of 95 °C for 10 s and 60 °C for 30 s, and finally a melt curve at 55–95 °C, every 0.5 °C lasting for 5 s. The ΔΔCt method was used to analyse the relative expression levels. The primers used for *AVP1*, *OsSIZ1* and *Fld* amplification were listed in Table S1.

### Plant propagation, maintenance and stress response assessment

To produce abundant plant materials for use in different stress experiments, the newly generated TG creeping bentgrass lines expressing all three genes, *AVP1*, *OsSIZ1*, *Fld* and those produced previously expressing only one of these three genes (Li *et al*., 2010, 2013, 2017), as well as the WT control plants, were clonally propagated from the same number of stolons and grown in small cone‐tainers (4.0 × 20.3 cm, Dillen Products, Middlefield, OH) or Elite 1200 pots (27.9 × 24.6 cm, Dillen Products, Middlefield, OH) using pure silica sand. All the plants were maintained as described previously (Li *et al*., 2010; Zhou *et al*., 2013). The plants were trimmed weekly to achieve uniform plant growth and were ready for stress treatment 10–14 weeks after maintenance in the growth room.

To evaluate plant salt stress response, the TG lines expressing all three transgenes, *AVP1, OsSIZ1* and *Fld* (TG1‐3) or only one of them, *AVP1* (Li *et al*., 2010), *OsSIZ1* (Li *et al*., 2013) or *Fld* (Li *et al*., 2017) as well as the non‐TG WT controls initiated from the same number of tillers were fully developed in cone‐tainers for 10 weeks under normal conditions in the growth room. Salinity stress was applied 1 week after the 10‐week‐old plants were uniformly clipped by watering plants every day with 10 mL of 200 ppm fertilizer (N‐P‐K, 20‐10‐20) supplemented with 0 or 200 mM NaCl, for 3 weeks. Plant performance was documented by photograph. The grass shoots and roots were harvested at the end of the stress, rinsed in Millipore (Billerica, MA) water, dried for 48 h at 80 °C, and measured for their DWs. The harvested materials were also used for measuring plant relative water content (RWC), electrolyte leakage (EL) and mineral contents.

To assess plant performance under drought stress, 10‐week‐old plants of different TG lines and WT controls were uniformly clipped and grown for one more week, and then subjected to drought stress by watering 15 mL of 200 ppm water‐soluble fertilizer (N‐P‐K, 20‐10‐20) once every 3 days for 2 weeks. The stressed plants were then fully watered daily with the 200 ppm 20‐10‐20 water‐soluble fertilizer for 2 weeks to allow plant recovery. The grass shoots and roots were harvested, rinsed and dried for 48 h at 80 °C to measure their DWs. The harvested materials were also used for measuring plant proline content.

To compare plant heat stress responses, three to ten replicates of both WT and various TG lines grown in cone‐tainers (4.0 × 20.3 cm, Dillen Products, Middlefield, OH) and Elite 1200 pots (27.9 × 24.6 cm, Dillen Products, Middlefield, OH), respectively, were transferred to the growth chamber (Conviron, Controlled Environments Inc., Pembina, ND) and maintained for 1 week under the same conditions as in growth room described above. Heat stress was then applied by heating the plants initially to 35 °C in the light and 30 °C in the dark for 1 week, and then at 40 °C in the light and 35 °C in the dark for another week. The relative humidity in the chamber was 60%–80% and the heat‐stressed plants were well‐watered every 2 days with 200 ppm fertilizer (N‐P‐K, 20‐10‐20). The cone‐tainers and pots with plants were placed in the 200 ppm fertilizer solution up to 4 cm from the bottom. Plant performance was documented by photograph and evaluated after the heat treatment and leaf EL was also measured.

To test plant performance under different nitrogen (N) supply concentrations, 12‐week‐old WT and TG plants grown in cone‐tainers with pure sand were mowed and grown for one more week, followed by thorough water flushing to remove residual nutrients from the plants and the sand. The plants were then nurtured using a modified 1 × Murashige and Skoog (MS) solution supplemented with N at the concentrations of 0.5 and 10 mM following the previously described procedure (Yuan *et al*., 2015). Three weeks after N treatment, leaf chlorophyll content was determined, and shoots and roots were harvested and dried for 48 h at 80 °C to measure DWs and plant.

To study plant response to phosphate (Pi) starvation, WT and different TG lines were grown in cone‐tainers with pure sand for 12 weeks, then uniformly trimmed and grown for one more week. The plants in sand were then thoroughly washed with water and nurtured every other day by a basal nutrient solution consisting of 1 × MS micronutrients and 1/10 × macronutrients without or with 10 μM Pi (H_2_PO_4_). Four weeks after treatments, leaf anthocyanin content was determined. Shoot and root biomass were measured after incubating at 80 °C for 48 h, and the total phosphorus in leaves and roots was also determined.

A completely randomized experimental design was used for all the stress response tests described above. We also randomly rotated all plant cone‐taners every other day during the experiment to minimize impact of a possibly heterogenous environment.

### Measurement of mineral content, N content, leaf RWC, EL, chlorophyll, proline and anthocyanin content

The minerals (Na^+^, K^+^, Ca^2+^, Mg^2+^) and soluble chloride contents in leaves and roots were determined using Spectro ARCOS ICP (Spectro, Mahwah, NJ) in Clemson University Agricultural Service Laboratory following protocols by Haynes (1980) and Plank (1992).

Plant total N content, leaf RWC, EL, chlorophyll, proline and anthocyanin content were measured according to previously published protocols (Bates *et al*., 1973; Dhindsa *et al*., 1981; Li *et al*., 2010).

### RNA sequencing, differential gene expression and GO enrichment analyses

Leaf total RNA was isolated from WT and the TG plants co‐expressing *AVP1*, *OsSIZ1* and *Fld* grown under normal conditions with Trizol reagent (Invitrogen) and used for cDNA library construction and paired‐end sequencing with DNBSEQ platform (100‐bp paired‐end reads) following the manufacturer's protocols (BGI‐America, San Jose, CA). Raw reads were mapped using RESM (Li and Dewey, 2011) and assigned using featureCounts (Liao *et al*., 2014). R 4.1.0 (R Core Team, 2022, https://www.R‐project.org) and the Bioconductor package edgeR (Robinson *et al*., 2010) were used to perform the differential gene expression analysis with FDR‐adjusted *P*‐value cut‐off of <0.05. The separation between WT and the TG samples and consistency between the three biological replicates in WT and the TG samples, respectively, were revealed by a PCA (principal component analysis) plot created using DESeq2 package (Love *et al*., 2014). EnhancedVolcano in R package was used to generate a volcano plot to illustrate log_2_ FC and the −log_10_ *P*‐value in the TG versus WT samples (Blighe *et al*., 2023). ComplexHeatmap package (Gu *et al*., 2016, http://www.bioconductor.org/packages/devel/bioc/html/ComplexHeatmap.html) was used to generate the heatmap based on the log_2_‐transformed count values to show gene expression profiles between WT and the TG samples. GO enrichment analysis was performed to gain information on the over‐represented functional categories. Since there is no GO annotation available for creeping bentgrass transcripts, putative GO terms were assigned using NCBI‐blast^+^ and Blast2GO 5.2 (Conesa *et al*., 2005; Liao *et al*., 2014).

### Statistical analysis

Summarized data (the counts, means and standard deviations for each group) were subjected to a one‐way ANOVA and the Tukey's HSD post hoc tests. Means not sharing the same letter are statistically significantly different (*P* < 0.05).

## Conflict of interest

The authors declare no conflict of interests.

## Author contributions

H.L. conceived and designed the experiments and supervised the research. Z.L. made the vectors for creeping bentgrass transformation. M.D., K.B., M.A., K.L., Q.H. and C.H. conducted creeping bentgrass transformation. G.Z., Y.L., L.L., R.C. and Z.L. carried out TG analysis to evaluate gene expression, plant development and plant stress responses. X.C., G.N. and H.L. performed bioinformatics analysis of the RNA‐seq data. G.Z., Y.L. and H.L. analysed the data and wrote the manuscript. All the authors participated in the discussion and revision of the manuscript.

## Supporting information


**Table S1** Primer pairs, amplicons, and transgene copy number determination in the transgenic lines COE1, 2 and 3.

## Data Availability

The data that support the findings of this study are available from the corresponding author upon reasonable request.
