# Supplementary material for: Gene pyramiding for boosted plant growth and broad abiotic stress tolerance
Source: Plant Biotechnol J. 2023 Oct 30;22(3):678–97. doi: 10.1111/pbi.14216 (PMC10893947; doi:10.1111/pbi.14216)
Supplement: Supplementary file 1 — Table S1 Primer pairs, amplicons, and transgene copy number determination in the transgenic lines COE1, 2 and 3. [file PBI-22-678-s001.docx]

**Supplementary Table 1.** Primer pairs, amplicons, and transgene copy number determination in the transgenic lines COE1, 2 and 3.

| Gene | Primer sequence (5’-3’) | Amplicon size | Melting curve peak | Primer pair efficiency | COE1 | COE2 | COE3 |
| --- | --- | --- | --- | --- | --- | --- | --- |
| *AsBRII* | AsBRI1_qF: GGTGGACTCGAAAACCTCGT  AsBRI1_qR: CTCCTTGGCTTCCTTGAGGG | 120 bp | 84 | 100% | 3 | 3 | 3 |
| *AVP1* | AVP1-qF: GTACTTACGACACCACCAGAAC  AVP1-qR: CATCCCAAGGAAACCAGATAGAA | 113 bp | 82.5 | 98% | 1 | 1 | 1 |

**Note:**

- The transgene copy numbers in the three independent hemizygous transgenic lines, COE1, 2 and 3 were determined by SYBR green based qPCR after Yuan et al. (2007) using Method 2, the internal reference gene-based method with a standard curve.
- The brassinosteroid receptor gene, *BRI1* employed to design internal standard curve experiments in this study was previously identified as a single-copy gene in rice (Saski et al., 2011). In creeping bentgrass, the presence and validation of three homeologs of the *BRI1* gene (*AsBRI1*) was confirmed by genome sequencing (Luo, unpublished).
- The gene-specific primer pairs targeting either all three copies of creeping bentgrass endogenous *AsBRI1* gene or the transgene *AVP1* from Arabidopsis were designed and are listed in Table S1.
- When determining gene copy number using qPCR method with the internal reference gene, the primer pair efficiency and its validation are imperative to ensure precise interpretation of data. Ideally, for a specific primer pair and template, the number of amplicons should double with each replication cycle, indicating a 100% amplification efficiency. To assess the efficiency of the *AsBRI1* and *AVP1* primer pairs, the wild type and transgenic creeping bentgrass total DNA (input per reaction: 0.01 ng, 0.1 ng, 1 ng, 10 ng and 100 ng) was used as the template in this experiment. The results showed that the efficiency of the *AsBRI1* and *AVP1* primer pairs was 100% and 98%, respectively (Table S1).
- The qPCR was conducted using the gene specific primers (Table S1) on an iCycler iQ system (Bio-Rad, Hercules, CA, USA) in 20 µl of PCR reaction solution (Luna® Universal qPCR Master Mix, New England Biolabs, MA, USA) with 2 nM of each primer. There were eight technical replicates for each of the three transgenic lines. PCR was conducted with the following program: an initial denature at 95°C for 120 s, followed by 40 cycles of 95°C for 15 s, 60°C for 30 s. Finally, a melting curve was performed from 65.0°C to 95.0°C at 0.5°C increment.
- The qPCR performed as described above allowed transgene copy number determination in all the transgenic lines generated in this research. The transgenic lines COE1, COE2 and COE3 (Table S1) from those having a single copy of transgene insertion were chosen as transgenic representatives for further analysis.

**References**

Saski, C.A., Li, Z., Feltus, F.A. Luo, H. (2011) New genomic resources for switchgrass: a BAC library and comparative analysis of homoeologous genomic regions harboring bioenergy traits. *BMC Genomics* **12**, 369. https://doi.org/10.1186/1471-2164-12-369.

Yuan, J.S., Burris, J., Stewart, N.R., Mentewab, A., Stewart Jr, C.N. (2007) Statistical tools for transgene copy number estimation based on real-time PCR. *BMC Bioinformatics* **8** (Suppl 7), S6. https://doi.org/10.1186/1471-2105-8-S7-S6.
